# Community structure of coral microbiomes is dependent on host morphology

**DOI:** 10.1186/s40168-022-01308-w

**Published:** 2022-07-28

**Authors:** Kathleen M. Morrow, M. Sabrina Pankey, Michael P. Lesser

**Affiliations:** 1 Department of Molecular, Cellular and Biomedical Sciences, University of New Hampshire, Durham, NH 03824 USA; 2 Present address: Thomas Jefferson High School for Science and Technology, 6560 Braddock Rd, Alexandria, VA 22312 USA

**Keywords:** Coral, 16S rRNA gene, Diazotroph, Microbiome, Nitrogen fixation, Symbiodiniaceae, *nif*H

## Abstract

**Background:**

The importance of symbiosis has long been recognized on coral reefs, where the photosynthetic dinoflagellates of corals (Symbiodiniaceae) are the primary symbiont. Numerous studies have now shown that a diverse assemblage of prokaryotes also make-up part of the microbiome of corals. A subset of these prokaryotes is capable of fixing nitrogen, known as diazotrophs, and is also present in the microbiome of scleractinian corals where they have been shown to supplement the holobiont nitrogen budget. Here, an analysis of the microbiomes of 16 coral species collected from Australia, Curaçao, and Hawai’i using three different marker genes (16S rRNA, *nif*H, and ITS2) is presented. These data were used to examine the effects of biogeography, coral traits, and ecological life history characteristics on the composition and diversity of the microbiome in corals and their diazotrophic communities.

**Results:**

The prokaryotic microbiome community composition (i.e., beta diversity) based on the 16S rRNA gene varied between sites and ecological life history characteristics, but coral morphology was the most significant factor affecting the microbiome of the corals studied. For 15 of the corals studied, only two species *Pocillopora acuta* and *Seriotopora hystrix*, both brooders, showed a weak relationship between the 16S rRNA gene community structure and the diazotrophic members of the microbiome using the *nif*H marker gene, suggesting that many corals support a microbiome with diazotrophic capabilities. The order *Rhizobiales*, a taxon that contains primarily diazotrophs, are common members of the coral microbiome and were eight times greater in relative abundances in Hawai’i compared to corals from either Curacao or Australia. However, for the diazotrophic component of the coral microbiome, only host species significantly influenced the composition and diversity of the community.

**Conclusions:**

The roles and interactions between members of the coral holobiont are still not well understood, especially critical functions provided by the coral microbiome (e.g., nitrogen fixation), and the variation of these functions across species. The findings presented here show the significant effect of morphology, a coral “super trait,” on the overall community structure of the microbiome in corals and that there is a strong association of the diazotrophic community within the microbiome of corals. However, the underlying coral traits linking the effects of host species on diazotrophic communities remain unknown.

Video Abstract

**Supplementary Information:**

The online version contains supplementary material available at 10.1186/s40168-022-01308-w.

## Background

Microorganisms in symbiosis with scleractinian corals form an intimate and dynamic relationship with their hosts, supporting several beneficial and potentially protective functions of ecological importance [1–3]. As part of the coral’s microbiome, specific groups of microorganisms are directly, or indirectly, involved in coral health and resilience when exposed to environmental stressors [1, 3]. Changes in particular microbes could act as bioindicators of environmental stress [1, 3, 4] and may impart pathogen resistance through the production of antimicrobial compounds [5, 6], the catabolism of dimethysulfoniopropionate (DMSP), and the production of sulfur-based antimicrobial compounds and antioxidants [7–10]. They are also critical to the acquisition and cycling of essential macro- and micronutrients such as carbon, nitrogen, phosphorus, trace metals, and vitamins [1, 3, 11–13]. As with all animal life [14], microorganisms have co-evolved with scleractinian corals and evidence for this can be observed at every life history stage from settlement through metamorphosis and development into an adult colony [15–19].

While the coral microbiome was generally believed to exhibit high diversity and host specificity [20], recent studies have shown that its composition can vary predictably based on host physiology and morphology [21], life history stage [16], and microhabitat within the mucus, tissues, and skeletal compartments of corals [1]. The composition of the coral microbiome also varies seasonally, geographically, and after exposure to different environmental (e.g., temperature, pollution, allelochemicals) conditions [13, 22–25]. As a result, and consistent with many other multipartite symbioses [26], the composition and function of the coral microbiome community are not static and appear to be influenced by host phylogeny, physiology, health, and the surrounding environment, on multiple spatial and temporal scales [1, 3]. There may also be specific low- or high-abundance bacterial or archaeal phylotypes that play a pivotal role in microbial stability [27], working together with other members of the microbiome to perform important functions, and further supported by functionally redundant members of the microbiome [1, 3].

One of these critical functions is the acquisition, transformation, and uptake of inorganic nitrogen, a limiting nutrient that corals generally obtain through the uptake of dissolved inorganic nitrogen by their algal symbionts or heterotrophic feeding by the host [28–31]. Nitrogen fixation is increasingly recognized as an essential component of the biogeochemical cycling of nitrogen and has also been measured within a variety of marine symbioses, including nitrogen-fixing bacteria and archaea (diazotrophs) living symbiotically with scleractinian corals [1, 32, 33]. Diazotrophs have been detected in the microbiomes of many coral species [34–38], and while rates of nitrogen fixation have been shown to be related to the abundance of diazotrophs [39], recent studies using general *nif*H primers have identified the need to identify and remove the non-nitrogen-fixing *nif*H clusters (i.e., clusters IV and V) from the analysis [40]. It is now believed that the acquisition of new nitrogen from the diazotrophic members of the coral microbiome may be important ecologically in the functioning of the coral holobiont [40–44]. Even more broadly, there is evidence suggesting a critical link between the susceptibility to coral bleaching and the availability and stoichiometry of environmental nitrogen and phosphorus [45–51].

Diazotrophy in corals has also been shown to increase the *in hospite* growth rates of Symbiodiniaceae without an increase in biomass [41]. Higher levels of nitrogen supplied by diazotrophic bacteria could release Symbiodiniaceae from nitrogen-limited growth and cause high rates of cell division and reduced translocation of photosynthates to the coral [52–54]. Stimulated diazotrophy due to elevated temperatures, from an increase in diazotrophic biomass or thermal effects on the kinetics of nitrogenase, is believed to be another mechanism by which the *in hospite* nutrient equilibrium of the coral holobiont becomes imbalanced, disrupting the N-limited state of Symbiodiniaceae and potentially inducing or prolonging bleaching events [47].

Given these emerging and ecologically important roles for diazotrophs in the biology of scleractinian corals, we present here a well-replicated multispecies microbiome study on corals from Australia, Curaçao, and Hawai’i. Specifically, we examined the bacteria and archaea (16S rRNA gene), diazotrophs (*nif*H), and Symbiodiniaceae (ITS2) compartments of each coral sample. With these data, and the phenotypic breadth of the samples collected, we asked if there were ecological or evolutionary phenotypes that determine the composition of the microbiome, with particular interest in diazotrophs, among different coral host taxa and locations.

## Methods

### Coral collection and site characterization

A total of 16 coral species (Supplemental Table 1) were sampled on SCUBA at a depth of 5–15 m from three locations: Tenements Reef northeast of Heron Island Research Station (HIRS; March 2015, *n* = 6 species, and *n* = 5–10 replicates per species), Australia (Lat: S 23° 26′ 33.7535″, Long: E 151° 54′ 54.5983″); Point Reef at the Hawai’i Institute of Marine Biology (HIMB; May 2015, *n* = 4 species, and *n* = 8–11 replicates per species), USA (21° 25′ 41.52″ N, 157° 47′ 30.84″ W); and Buoy 1 reef near the Caribbean Marine Biological Institute (CARMABI; March 2016, *n* = 7 species, and *n* = 6 replicates per species), Curaçao (12° 7′ 28.65″ N, 68° 58′ 23.23″ W). These collections constitute a “natural experiment” where coral species exhibiting a range of coral traits and life history characteristics (Supplemental Table 1) were collected under similar conditions (e.g., irradiance) but from different locations. For coral collections, divers wore nitrile gloves to collect samples for microbial analyses and coral branches were clipped with bone cutters and placed in sterile Whirl-Pak® bags (Nasco). In addition to coral nubbins, seawater (*n* = 5–8 per site × 1 l using Nalgene® collapsible plastic containers that were acid washed and rinsed with Milli-Q® water) and porewater (*n* = 5 per site) where ~ 25-30 ml of sediment adjacent to corals was collected using 50 ml tubes, allowed to settle,, and the seawater supernatant sampled. These samples were returned to the laboratory where they were each immediately filtered onto a GF/F filter (0.7 μm but see [55] for better filtration performance than stated by the manufacturer), and placed in a 2-ml cryovial with a DNA preservation buffer [56] and stored at −20°C. The temperature (°C) and irradiance (*E*_d_) of photosynthetically active radiation (PAR: 400–700 nm), reported as μmol quanta m^−2^ s^−1^, were taken hourly over several days during the sample collection period using HOBO Pendant Temperature/Light loggers (*n* = 3 per site), measurements of irradiance were in lumens m^−2^ and converted to quanta [57] by calibrating against a LiCor cosine-corrected, planar, sensor (LI 192SA). Water samples for nutrient concentrations (i.e., NOx) were collected from ~15 m as previously described [40].

### Coral processing and gDNA extraction

Coral tissues were rinsed with sterile 1× phosphate-buffered saline (PBS) using an airbrush at a distance of 10–20 cm for ~ 30 s on corals hanging upside down to remove as much coral mucous as possible with its associated microbes and other cellular debris. Coral tissues were then removed from the skeleton using pressurized air (~130–150 psi) from a blow gun with sterile tips into a sterile bag (Whirlpak®, Nasco). The blow-gun tip was wiped clean with 70% ethanol followed by sterile 1× PBS between each sample. The tissue blastate was homogenized for 30 s at medium speed using a hand-held, variable speed, tissue homogenizer (BioSpec, Tissue-Tearor®) which was run in 70% ethanol followed by sterile 1× PBS between each sample. This coral homogenate was then separated into the host, Symbiodiniaceae, and bacterial fractions as follows: the coral homogenate containing coral tissue, Symbiodiniacaea, bacteria, and skeletal debris was gently pelleted (400 × *g*, 5 min, 4°C). From this homogenate, a 2-ml aliquot of the bacteria-enriched supernatant was removed and pelleted in a fixed-angle centrifuge (20,000 × g, 10 min). This cell pellet was fixed in DNA buffer [56] and used for 16S rRNA and *nif*H gene analysis and stored at −20°C as the bacterial fraction which would include bacteria from the host, the skeletal debris, and the phycosphere of the Symbiodiniaceae. The remaining homogenate was remixed and an aliquot (~2 ml) containing the coral tissue and Symbiodiniaceae cells was pelleted (4000 × *g*, 10 min, 4°C). The supernatant containing the host fraction was discarded and the pellets were remixed in 0.02% sodium dodecyl sulfate (SDS) in 1× PBS at room temperature and incubated for 30 min followed by three additional rinses with 1× PBS (4000 × *g*, 10 min, 4°C) to remove any residual SDS. After the last rinse, a 2-ml sample of cleaned Symbiodiniaceae cells was collected, pelleted (4000 × *g*, 10 min, 4°C), preserved in 2 ml of DNA buffer, and stored at −20°C for ITS2 gene analysis as the Symbiodiniaceae fraction. For the Symbiodiniaceae fraction, no analysis using the 16S rRNA and *nif*H genes was conducted to capture the endosymbionts of the algal cells in this study.

Genomic DNA (gDNA) was isolated from the preserved bacterial fractions described above, and the seawater and porewater filters using the MOBIO PowerSoil® DNA isolation kit following the manufacturer’s instructions with the addition of a 10-min 65°C heating step prior to two 2-min bead beating using a Qiagen QuickLyser set at 50 Megahertz for the bacterial analyses*.* Genomic DNA was extracted from Symbiodiniaceae fractions using the MOBIO PowerPlant® DNA isolation kit with modifications as previously described [58]. All bacterial and Symbiodiniaceae gDNA was checked for quality and concentration using a NanoDrop spectrophotometer 2000c.

### Amplification of microbial 16S rRNA gene, nifH, and dinoflagellate ITS2 genes

A total of 164 coral and environmental samples (Supplemental Table 2) were polymerase chain reaction (PCR)-amplified with two primer sets to target the universal bacterial/archaeal 16S rRNA gene and nitrogenase gene (i.e., *nif*H). Linker primer sequences CS1 (5′ - ACACTGACGACATGGTTCTACA) and CS2 (5′ - TACGGTAGCAGAGACTTGGTCT) were added to the 5′ end of both forward and reverse primers to facilitate sequencing. The 16S rRNA gene was amplified from the bacterial pellet using updated Earth Microbiome degenerate primers designed to amplify the hypervariable region V4, consisting of the forward primer 515F (5′ - GTGYCAGCMGCCGCGGTAA [59]) and the reverse primer 806RB (5′ - GGACTACNVGGGTWTCTAAT [60]). The 16S rRNA gene PCR consisted of a 25-μl reaction with 12.5 μl AmpliTaq Gold® 360 Master Mix (Applied Biosystems), 1.0 μl GC-enhancer, 0.5 μl 515F (10 μM) and 0.5 μl 806RB (10 μM), 2.0 μl of DNA template (100–150 ng), and 8.5 μl nuclease-free water (Integrated DNA Technologies, Coralville, Iowa). Reactions were performed using the Earth Microbiome protocol: initial denaturation for 10 min at 95°C, 30 cycles of 95°C for 45 s, 50°C for 60 s, and 72°C for 90 s, followed by a 10-min extension at 72°C.

All coral (i.e., bacterial pellet) and environmental samples (i.e., seawater and sediment porewater) were amplified with the *nif*H gene-specific primers IGK3 (5′ - GCIWTHTAYGGIAARGGIGGIATHGGIAA) and DVV (5′ - ATIGCRAAICCICCRCAIACIACRTC [61]). These primers capture both phylotypes of the non-nitrogen-fixing clusters IV and V and nitrogen-fixing clusters I and III. The *nif*H PCR consisted of a 25-μl reaction with 12.5 μl AmpliTaq Gold® 360 Master Mix (Applied Biosystems), 1.5 μl GC-enhancer, 3.0 μl IGK3 (10 μM) and 3.0 μl 806RB (10 μM), and 5.0 μl of DNA template (40–60 ng). Reactions were performed using the following protocol: initial denaturation for 10 min at 95°C, 40 cycles of 95°C for 45 s, 57°C for 45 s, and 72°C for 60 s, followed by a 10-min extension at 72°C. Samples were purified with Agencourt AMPure XP bead kit (Beckman Coulter, Danvers, MA) to remove primer dimers followed by sequencing.

The Symbiodiniaceae samples (i.e., cleaned algal pellet) were amplified with ITS2-Dino-F (5′ - GTGAATTGCAGAACTCCGTG [62]) and its2rev2 (5′ - CCTCCGCTTACTTATATGCTT [59]). The ITS2 PCR consisted of a 25-μl reaction with 12.5 μl AmpliTaq Gold® 360 Master Mix (Applied Biosystems), 1.0 μl GC-enhancer, 0.5 μl ITS-Dino-F (10 μM) and 0.5 μl its2rev2 (10 μM), 8.5 μl nuclease-free water (IDT), and DNA template (40–60 ng). Reactions were performed using the following protocol modified from Stat et al. [63]: initial denaturation for 10 min at 95°C, 30 cycles of 95°C for 45 s, 52°C for 45 s, and 72°C for 45 s, followed by a 7-min extension at 72°C.

All PCR products described above, including negative kit and PCR controls, were electrophoresed on a 1% agarose gel for screening, and amplified samples were submitted to the University of Illinois at Chicago Research Resources Center for sequencing.

### Sequencing and bioinformatics

Sequencing was performed using two PCR steps to generate PCR amplicons ready for sequencing on an Illumina MiSeq sequencer. The first PCR was performed as described above. A second 8-cycle reaction was performed using new primers that target the common sequences and contain Illumina sequence adapters and sample-specific barcodes (10 bases). Samples were purified using SequalPrep™ Normalization Plates (Invitrogen, Carlsbad, CA) to remove primer dimers and other non-specific DNAs present in the sample and to normalize DNA concentration. The purified amplicons were pooled, and the final pool was quantified and loaded on the Illumina MiSeq sequencer with a flow cell employing V2 chemistry with Symbiodiniaceae ITS2 primers (2 × 250 bp reads, ~10,000–20,000 reads per sample) and V3 chemistry with 16S rRNA and *nif*H primers (2 × 300 bp reads, ~20,000–40,000 reads per sample).

A total of 1.04 × 10^7^ demultiplexed paired-end 16S rRNA gene sequences were imported into Quantitative Insights into Microbial Ecology (QIIME2 v2018.2) [64]. Reads were filtered for quality and chimeric sequences using DADA2 with standard parameters (maxN=0, truncQ=2, rm.phix=TRUE, and maxEE=2) [65], and taxonomic classification was assigned using a naïve Bayes classifier trained on the extracted region of interest from the SILVA 16S rRNA gene reference (Release 132) alignment [66]. All sequences classified as chloroplast or mitochondria were removed. The resulting amplicon sequence variant (ASV) table was used for statistical analysis. A PhyloSeq [67] object was constructed in R from the QIIME2 generated feature table with 44,895 identified ASV’s and associated metadata. Prior to statistical analyses, samples were filtered using PhyloSeq by both prevalence (ASVs detected in at least 2 samples and accounting for at least 10 occurrences across samples), which dropped the total identified ASVs to 4244. Samples were then rarefied to the smallest library size (3400 counts) to normalize for sequencing effort. Data were transformed using the Centered Log Ratio (CLR) [68], and a Euclidean distance matrix (i.e., Aitchison distance) was built to examine patterns of community structure between groups of samples. Principal coordinate analyses (PCoA) with Pearson correlation vectors were further used to visualize community structure and examine which ASVs have strong positive or negative correlations with either PCO axis, indicative of sample separation.

The effect of coral species on microbial alpha diversity (Shannon index, using the diversity() function in “vegan” R package [69]) was tested using the Kruskal-Wallis rank-sum test on raw counts following rarefaction. To account for variance due to coral species while testing other factors, a nested ANOVA approach was employed by fitting mixed models using the lme() function in the “nlme” R package [70]. Model terms were fit by maximizing the restricted log-likelihood (i.e., REML method). Factors such as sampling site and coral traits and life history characteristics (Supplemental Table 1, https://coraltraits.org/traits/) were each fitted as fixed effects and coral species included as a nested random effect. Single intercept and random slope models were compared using ANOVA. Tukey’s multiple comparison tests were performed using the glht() function from the R package “multcomp” [71]. To control for species differences while testing for composition effects (beta diversity) of each of the other factors (sampling site and coral traits), a nested PERMANOVA approach with 999 permutations was employed using the nested.npmanova() function from the “BiodiversityR” R package [72]. The abundances of *Rhizobiales* were assessed for differences across coral factors using the same nested PERMANOVA approach described above, on raw counts after rarefaction followed by post hoc Tukey’s HSD multiple comparison tests. The taxonomic status of *Rhizobiales*, while still valid, does not follow the rules of nomenclature of the International Code of Nomenclature of Prokaryotes (ICNP). The order *Rhizobiales* has been renamed *Hyphomicrobiales* based on full-length 16S rRNA sequences [73] but remains unapproved by the ICNP. We retained the use of the nomenclature *Rhizobiales* for most of the analyses in this study. To assess the effects of coral traits on ASV enrichment, Wald significance tests were evaluated on raw count data with the function DESeq() from the “DESeq2” R package, with size factors estimated from the geometric means of the counts. Only ASVs obtaining adjusted *p* values < 0.05 were reported.

The *nif*H reads were oligotyped using Minimum Entropy Decomposition (MED) as implemented in the TaxADivA (TAXonomy Assignment and DIVersity Assessment) pipeline, with the settings: (-y -k -r 26 -l 29 --keepc4 -g 500). Briefly, forward and reverse reads were merged using PEAR (--pear "-v 20 -m 450 -n 300 -p 1.0 -j 12") [74] and any merged reads less than 300 bp or greater than 450 bp were discarded [64]. Reads were trimmed using PRINSEQ (left: 29, right: 26 [75]), chimeras removed with VSEARCH [76], and any libraries with fewer than 500 reads were discarded. Reads were clustered into novel oligotypes using MED [77] and the reference *nif*H database provided with TaxADivA. Following MED analysis, novel oligotypes were clustered at 95% similarity using cd-hit-est (CD-HIT suite) [78] and then translated to peptides using TransDecoder (http://transdecoder.github.io/), predictions following blastp queries against NCBI *nif*H peptide representatives. Peptide translations for de novo *nifH* and best-match NCBI *nif*H sequences were aligned with MAFFT along with nitrogenase-like chlorophyllide reductases and ferrodoxins [79]. An initial maximum likelihood topology was constructed with RAxML [80], using the PROTGAMMAAUTO method to fit a model of protein substitution. De novo *nif*H sequences within the chlorophyllide/ferrodoxin clades (i.e., Clades IV and V) were excluded from the analysis. Validated de novo *nif*H sequences were then queried against nr using Blastp (to the exclusion of unculturable/environmental samples). These matches were then aligned with the refined *nif*H dataset using MAFFT before final phylogenetic inference. To ascertain node support, bootstraps were conducted under the best-fit model (PROTGAMMALG) until the MRE convergence criteria were satisfied (-I autoMRE). Only *nif*H phylotypes with phylogenetic relationships to established nitrogen-fixing *nif*H clades (e.g., clusters I and III, bootstrap support >90%) were subsequently analyzed. All samples were subjected to rarefaction (Supplemental Fig. 1) to normalize for sequencing depth and only samples with at least 500 cluster I and III reads were analyzed. Alpha and beta diversity analysis of *nif*H phylotypes, using nested PERMANOVA, followed the same approach as described above for 16S rRNA gene ASVs.

A co-inertia analysis (CIA) using the function cointertia() from the R package “made4” [81] was conducted to identify trends and co-relationships between the 16S rRNA gene and *nif*H marker gene datasets. CIA is a multivariate method that identifies the strength of association among datasets [82]. To measure the overall similarity for the marker genes, an RV coefficient is calculated for each factor (e.g., species, site, coral traits) using a Monte Carlo test on the sum of eigenvalues with the function RV.rtest() in the R package “ade4” [83]. The RV coefficient is a multivariate generalization of the squared Pearson correlation coefficient that measures the closeness of two sets of points that may each be represented in a matrix.

For Symbiodiniaceae communities, the ASVs were analyzed and tabulated across samples using DADA2 [65]. Briefly, raw reads were trimmed of the initial 15 bp (forward) and 80 (reverse) to remove residual primer and low-quality bases. The tail ends of reads were trimmed during quality control (15 bp forward, 120 bp reverse). All reads were truncated beyond the first instance of quality scores below 3 (truncQ = 2). The maximum expected error during denoising (maxEE) was 2 for all reads. Denoised reads were then merged and chimeric contigs discarded using mergePairs and removeBimeraDenovo, respectively. Symbiodiniaceae taxonomy was then assigned to each ITS2 ASV using a BLAST query against a Symbiodiniaceae ITS2 database implemented in SymITS2 (https://github.com/jrcunning/SymITS2). ASVs with ≥ 95% sequence similarity to reference Symbiodiniaceae were retained and assigned to a Symbiodiniaceae genus as well as to the best matching ITS2 sequence accession (or cluster of identical accessions). Genotypes were identified to species following LaJeunesse et al. [84]. Only libraries of more than 10,000 read counts were retained for analysis. An average of 2.9 × 10^4^ (± 4.9 × 10^3^ SD) reads per sample were genotyped with SymITS2 following quality filtering and chimera removal, yielding 630 distinct ASVs with matches to 150 published genotypes across the genera *Breviolum*, *Cladocopium*, *Durusdinium*, and *Symbiodinium* prior to rarefaction.

Subsequently, the analyses of the Symbiodiniaceae community composition were carried out using PhyloSeq functions in R [67]. The ASV count table was filtered to include ASVs detected in at least 2 samples and accounting for at least 10 occurrences across samples. Samples were then rarefied to the smallest library size (17,476 counts) to normalize for sequencing effort. Alpha diversities were estimated using the Shannon index. Samples were ordinated based on Bray-Curtis distance using principal coordinates analysis (method = “PCoA”). To assess compositional differences across traits, the rarefied ASV count table was consolidated by algal genus or best ITS2 hit using the “tax_glom” function. Raw counts were transformed to center log ratios using the “transform” function (transform = “CLR”) from the R “microbiome” package [85]. Compositional differences among coral species were then tested using PERMANOVA on sample Aitchison distances [68] with the “adonis” function from the R package “vegan” [69]. Nested PERMANOVAs were used to test site and trait effects on ITS2 composition while controlling for species effect as described earlier. Correlation between 16S and ITS2 community compositions was evaluated using a Mantel test on Aitchison distances with the “mantel” function from vegan [69].

## Results

Temperature, irradiance, and NOx concentrations were previously published for the coral collection sites [40, 42] and are briefly summarized here. At the time of coral collection in Curaçao, the ambient seawater temperature was 27.3°C ± 0.2 (SD); in Kaneohe Bay, Hawai’i, it was 25.4°C ± 0.5 (SD); and in Australia, it was 27.9°C ± 0.6 (SD). Seawater temperatures were statistically different between sites (ANOVA *F*_2,8_ = 23.6, *P* = 0.001) with temperatures in Hawai’i significantly lower (Tukey’s HSD, *P* < 0.05) than Curaçao or Australia which were not significantly different from each other (Tukey’s HSD, *P* > 0.05). Downwelling irradiance (*E*_d_) at solar noon was 560 ± 86 (SD) μmol quanta m^−2^ s^−1^ in Curaçao; in Kaneohe Bay, it was 620 ± 73 (SD) μmol quanta m^−2^ s^−1^ ± 73; and in Australia, it was 475 ± 74 (SD) μmol quanta m^−2^ s^−1^. Irradiances were not statistically different between sites (ANOVA *F*_2,8_ = 2.6, *P* = 0.152). The concentration of NOx (NO_3_^−^ and NO_2_^−^) in seawater was 0.83 μmol N l^−1^ ± 0.2 (SD) in Curaçao; for Hawai’i, it was 1.8 μmol N l^−1^ ± 0.3 (SD); and in Australia, it was 0.71 μmol N l^−1^ ± 0.5 (SD). NOx concentrations were statistically different between sites (ANOVA *F*_2,8_ = 34.6, *P* = 0.0005) with significantly higher concentrations in Hawai’i (Tukey’s HSD, *P* < 0.05) than Curaçao or Australia which were not significantly different from each other (Tukey’s HSD, *P* > 0.05). The temperature and nutrient differences reflect the differences in reefs where Hawai’i is a tropical estuary with significant terrestrial influence and Curaçao is a coastal system also influenced by riverine discharge whereas Heron Island in Australia is an offshore reef. Microbial diversity among seawater samples did vary significantly between all sites, in terms of both beta diversity (PERMANOVA *F*_2,15_ = 6.7, *P* < 0.001) and alpha diversity, with HIRS displaying the greatest Shannon diversity and CARMABI the lowest (ANOVA *F*_2,15_ = 20.5, *P* < 0.001). However, microbial alpha diversity among coral samples also did not differ significantly with respect to site, when controlling for the effect of species (nested ANOVA *F*_2,14_ = 1.54, *P* = 0.249).

For most of the traits and life history characteristics of corals (Supplemental Table 1), there are > 3 replicates, but there are limited representatives for some traits (e.g., solitary morphologies). Alpha diversity (i.e., within-sample diversity) based on the 16S rRNA gene using the Shannon diversity index, including potential diazotrophs, varied significantly among coral species (Kruskal-Wallis 𝛸^2^_(16)_ = 96, *P* << 0.001) but not reproductive strategy (nested ANOVA *F*_4,12_ = 0.81, *P* = 0.542), ecological strategy (nested ANOVA *F*_3,13_ = 1.95, *P* = 0.171), phylogenetic clade (nested ANOVA *F*_1,15_ = 3.47, *P* = 0.082), or spawning mode (nested ANOVA *F*_1,15_ = 2.4, *P* = 0.14).

**Fig. 1:**
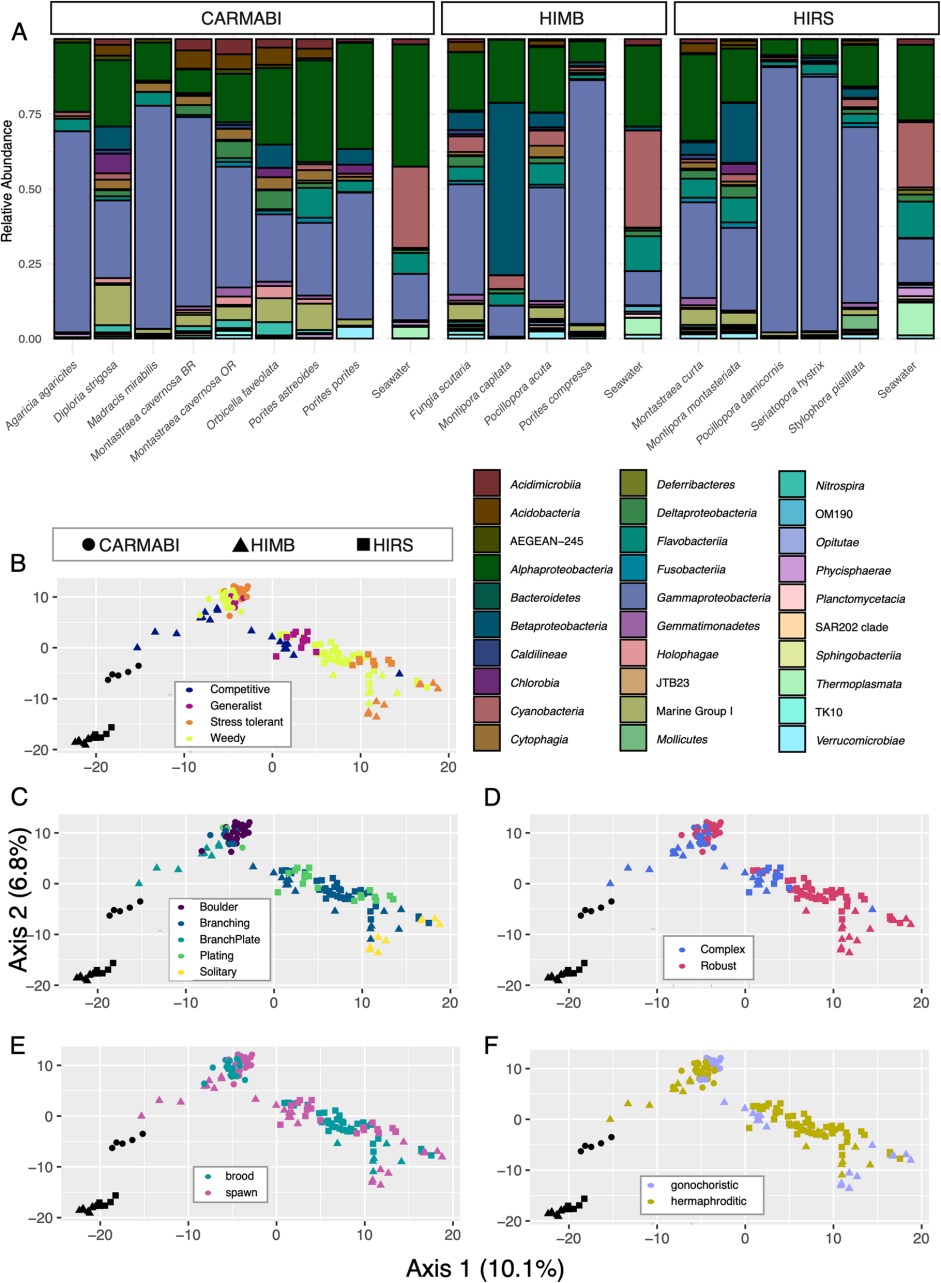
Estimates of beta diversity from 16S rRNA gene microbiome communities across coral species. **A** Relative abundances of microbial classes averaged for each coral species. Note that only microbial classes representing > 1% of all reads were included for visualization. Principal coordinates of coral samples colored by coral life history traits based on 16S rRNA gene microbial composition for **B** ecological life history characteristics, **C** morphology, **D** phylogenetic clade, **E** spawning mode, and **F** reproductive strategy. Significance testing was based on nested PERMANOVA analyses to account for variation due to coral species, which was highly significant (Table 1). Seawater samples are shown in black and sampling location is denoted by shape

**Fig. 2:**
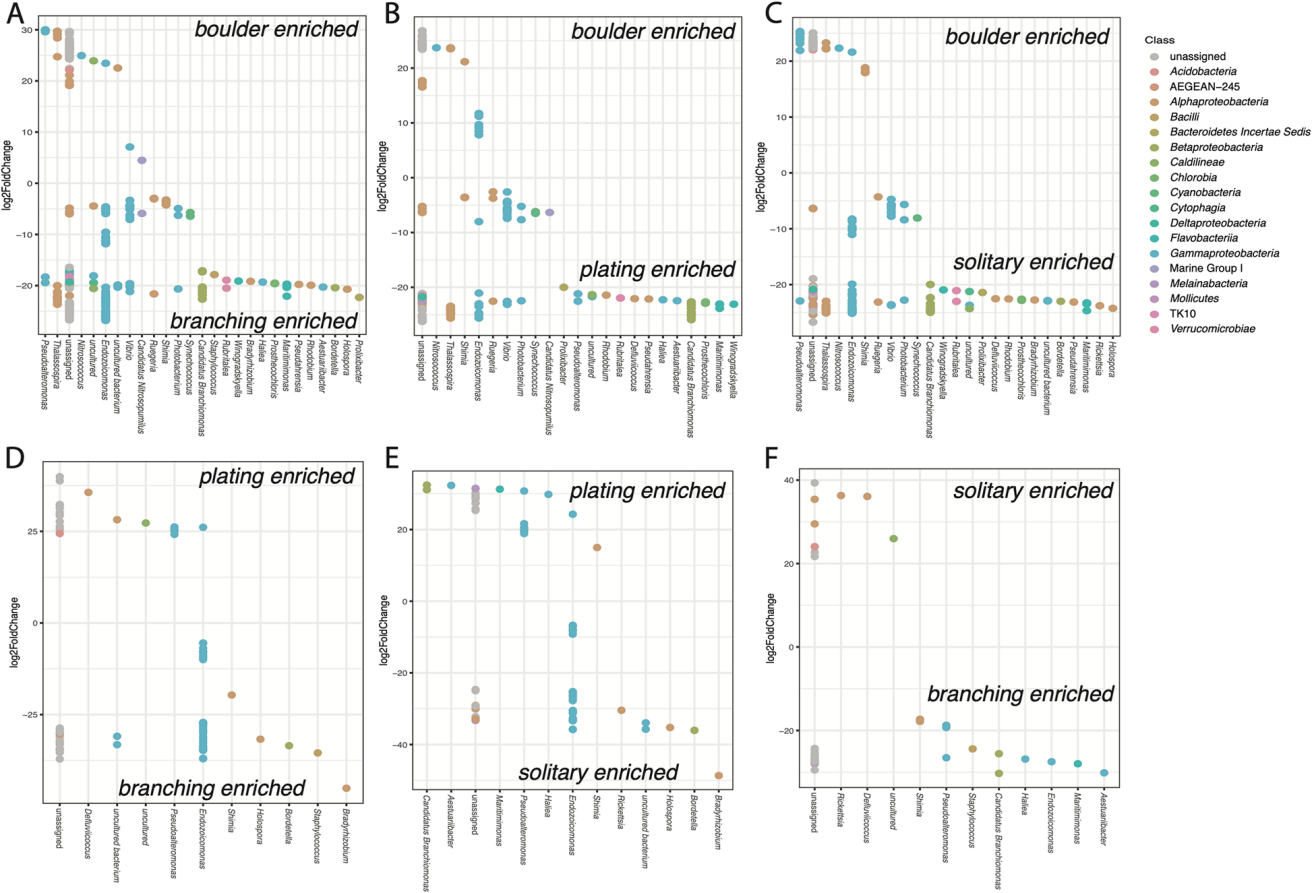
ASVs with differential enrichment with respect to coral morphology. Significant ASVs determined using Wald tests, adjusted *P* values < 0.05. **A** Boulder vs branching; **B** boulder vs plating; **C** solitary vs boulder; **D** plating vs branching; **E** solitary vs plating; **F** branching vs solitary. Relative enrichment (log2 scale) shown for ASVs, grouped by assigned genus and colored by microbial class

Significant effects on the microbiome community composition, based on 16S rRNA gene sequences at both the ASV and phylum levels were detected for coral species (Fig. 1A), site, phylogenetic clade, and ecological life history characteristics (Fig. 1). When controlling for species using a nested PERMANOVA, however, significant effects on phyletic composition were observed for site, morphology, and ecological life history characteristics (Table 1). Among these factors, morphological differences showed the most significant effect on microbial community composition (Fig. 1C). Prokaryotes with relative abundances that differed significantly among coral species belonged predominantly to *Alpha*- and *Gammaproteobacteria*, with *Endozoicomonas* accounting for 23% of variably enriched ASVs (Supplemental Table 3, Figs. S2-S4). Among sites, CARMABI samples were relatively enriched in *Pseudoalteromonas*, while HIMB and HIRS were both enriched for members of the *Endozoicomonas* and *Delftia*. HIMB was also enriched for a member of the *Bradyrhizobium* (Supplemental Fig. 2). Coral traits and life history characteristics varied in their effect on ASV enrichment, with site and morphology affecting the largest number of ASVs and reproductive strategy affecting the fewest ASVs. Corals with a boulder morphology consistently yielded large numbers of ASVs that were significantly depleted relative to other coral morphologies. For example, branching, plating, and solitary corals all demonstrated more than 110 ASVs with greater differential abundance than boulder morphologies (Fig. 2). These differences spanned 18 microbial classes and accounted for more diversity than observed for ASV differences between other morphologies, with the bulk of ASVs identified as *Gammaproteobacteria* (e.g., *Endozoicomonas*). When the reproductive strategy was examined, hermaphroditic corals were enriched for seven *Endozoicomonas* ASVs compared to gonochoristic samples (Supplemental Fig. 3A). Broadcast spawning corals were relatively more diverse, showing enriched levels of several ASVs of *Gammaproteobacteria* (e.g., *Endozoicomonas*) and *Betaproteobacteria* (e.g., *Delftia*) in comparison to brooding corals (Supplemental Fig. 3B). Robust clade corals were enriched for several *Gammaproteobacteria* ASVs (e.g., *Endozoicomonas*) compared to complex corals (Supplemental Fig. 3C). Among ecological life history strategies, competitive species were consistently enriched for *Delftia* ASVs in the *Betaproteobacteria* compared to weedy, stress-tolerant, or generalist strategies (Supplemental Fig. 4). Looking at life history characteristics, both competitive and generalist species yielded the greatest differences in terms of differentially enriched ASVs (*n* = 76), with competitive corals enriched for more *Endozoicomonas* ASVs and generalists enriched for more *Pseudoalteromonas* ASVs. Weedy and stress-tolerant species differed the least, with only 21 ASVs differentially enriched.

**Table 1:** Nested PERMANOVA result summary for effects of site and coral traits on 16S rRNA gene community composition, controlling for species effect. Predictor variables were tested on all count data (3228 ASVs) and with counts aggregated by phylum (27 phyla). Coral traits: morphology (mounding, branching, plating, solitary); reproductive strategy (gonochoristic or hermaphroditic); spawning mode (brooding, spawning); phylogenetic clade (robust, complex); ecological life history characteristics (weedy, competitive, generalist, stress-tolerant). Standard PERMANOVA for species effect alone is provided in the last row

		*ASV*		*Phylum*	
*Factors*	*df*	*F*	*P*	*F*	*P*
Site	2, 117	1.99	0.002	1.98	0.04
Morphology	3, 117	1.65	0.004	2.69	<0.001
Reproductive strategy	2, 117	0.91	0.654	1.30	0.28
Spawning mode	1, 117	1.3	0.18	2.0	0.06
Phylogenetic clade	1, 117	1.53	0.072	1.50	0.18
Ecology	3, 117	1.17	0.215	2.01	0.02
Species	16, 117	5.6	<0.0001	4.5	<0.0001

**Fig. 3:**
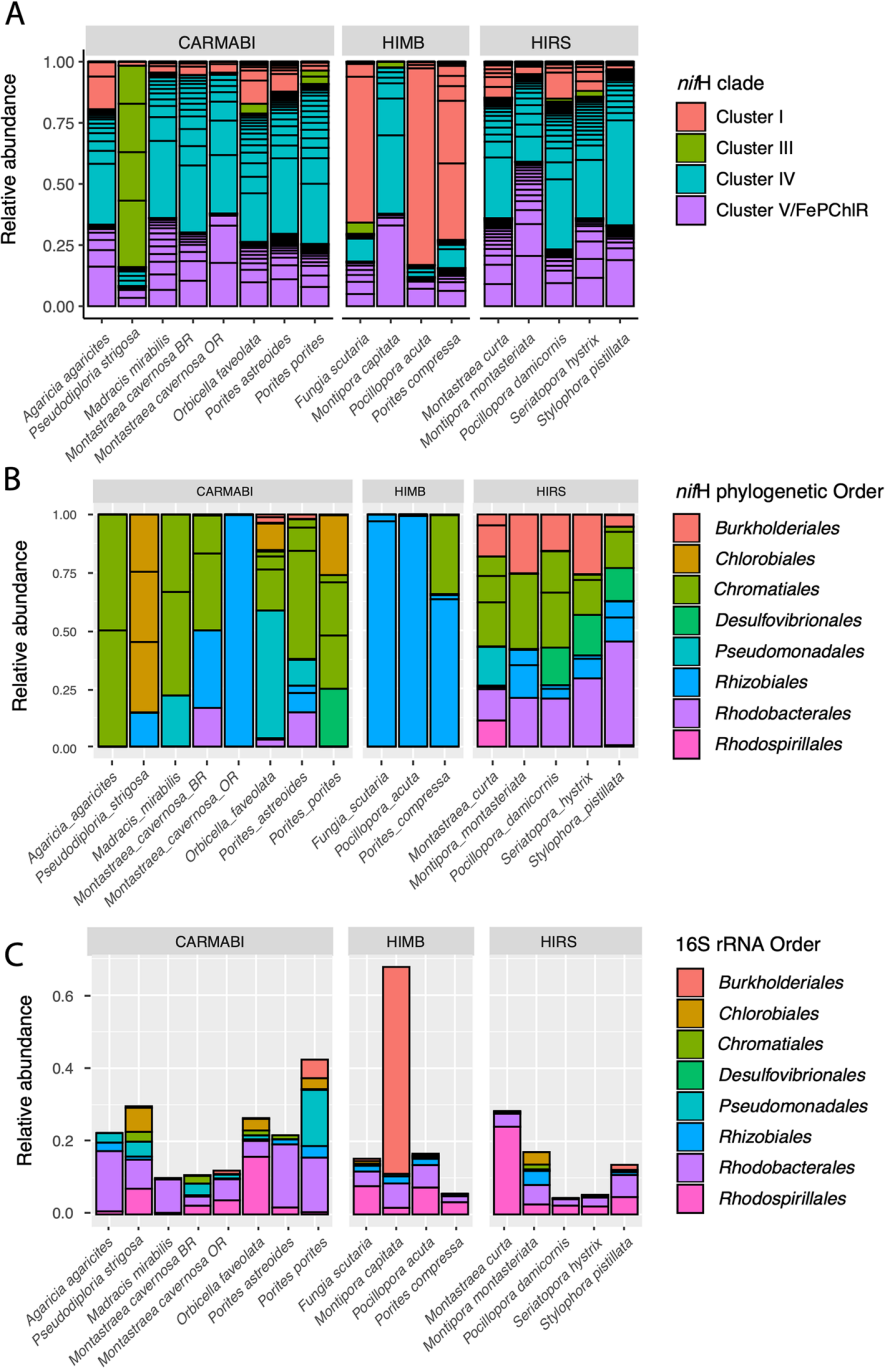
**A** Relative abundance of each *nif*H phylotype (unique sequence variant) according to placement in either nitrogen-fixing or non-nitrogenase clades. Average abundances calculated after combining samples for each species. **B** Relative abundance of nifH phylotypes according to their nitrogen-fixing orders. Average abundances calculated after combining samples for each species and only include variants belonging to *nif*H clusters I and Ill. **C** Relative abundance of 16S rRNA orders containing candidate diazotrophs. Average abundances calculated after combining samples for each species

For the *nif*H gene sequences, representatives of all the non-nitrogen-fixing (clusters IV, V: 80 phylotypes) and nitrogen-fixing clades (clusters I, II, and III: 25 phylotypes) were recovered (Fig. 3A). In particular, the non-nitrogen-fixing ASVs made up a majority of reads across all coral species (Fig. 3A) as previously reported for corals [40]. Of note, *Rhizobiales* were recovered in the majority of (i.e., 11 of 16 species across regions) corals examined (Fig. 3B). We excluded *Montipora capitata* from the Hawai’i *nif*H analyses because these samples had < 500 cluster I/III reads. For those 16S rRNA gene taxa identified as putative diazotrophs, with the caveat that not all of these will be nitrogen fixers, an ANOVA showed that coral species varied significantly in terms of the 16S rRNA gene relative abundance of these diazotrophic candidates (Fig. 3C). However, most coral traits were not associated with a change in the relative abundances of these diazotrophic candidates, except for ecological life history characteristics in the orders *Desulfovibrionales* (generalist > competitive, nested ANOVA *F*_3,13_ = 3.5, *P* = 0.048) and *Burkholderiales* (competitive > stress and weedy, ANOVA *F*_3,13_ = 3.7, *P* = 0.041). Morphology was correlated with a change in the relative abundance of *Burkholderiales* (branching and plating > boulder, ANOVA *F*_4,12_=129.0, *P* < 0.001).

**Fig. 4:**
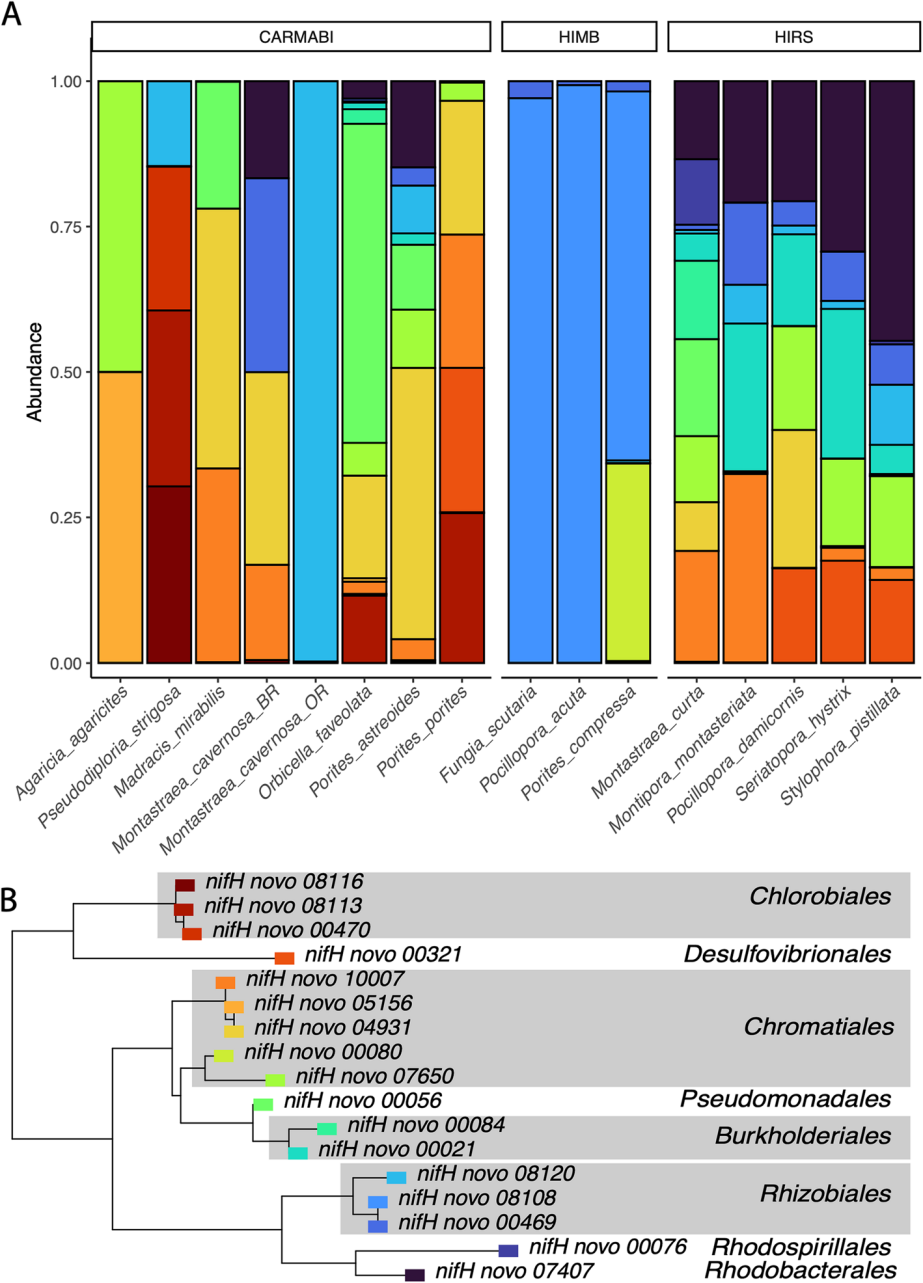
*nif*H diversity across coral species. **A** Average relative abundances of *nif*H phylotypes (unique sequence variants) belonging to nitrogen-fixing clusters I and III. Bar segments are colored according to placement in **B** phylogeny of *nif*H amplicons. Peptide translations for de novo *nif*H and best-match NCBI *nif*H sequences were aligned with MAFFT along with nitrogenase-like chlorophyllide reductases. De novo *nif*H that placed within the chlorophyllide/ferrodoxin clades were excluded. Only *nif*H phylotypes with phylogenetic relationships to established nitrogen-fixing *nif*H clades (e.g., clusters I and III) were subsequently analyzed. Taxonomic affinity of *nif*H variants is based on phylogenetic placement using published sequences. Note: only phylotypes representing at least 1% of all reads were included

The analysis of nitrogen-fixing *nif*H phylotypes (clusters I and III) showed that both coral species and sampling site (Fig. 4A) had significant effects on the alpha (Kruskal-Wallis 𝛸^2^_(15)_ = 55, *P* << 0.001) and beta diversity (PERMANOVA *F*_15,89_ = 5.94, *P* = 0.001) for *nif*H phylotypes (Fig. 4B). For sampling sites, only HIMB and HIRS showed significant differences in *nif*H alpha diversity, after controlling for the variation due to species using post hoc multiple comparison (Tukey’s HSD: *P* < 0.05). There was no effect of spawning mode on alpha diversity (nested ANOVA *F*_1,14_ = 1.7, *P* = 0.22), while reproductive strategy showed that gonochoristic corals had a greater relative abundance of *Rhizobiales* phylotypes (nested ANOVA, RS: *F*_1,14_ = 4.72, *P* = 0.047). Complex corals harbored a greater relative abundance of *Chromatiales* (nested ANOVA *F*_1,14_ = 4.8, *P* = 0.044) than robust corals. The site was also correlated with changes in the relative abundance of several diazotrophic groups in the *nif*H dataset compared to the 16S rRNA gene dataset. Specifically, HIRS showed higher relative abundances of *Rhodobacterales* (nested ANOVA *F*_2,13_ = 11.7, *P* = 0.001) and *Burkholderiales* (nested ANOVA *F*_2,13_ = 9.9, *P* = 0.002), while HIMB was correlated with a greater relative abundance of *Rhizobiales* (nested ANOVA *F*_2,13_ = 24.5, *P* < 0.0001).

**Fig. 5:**
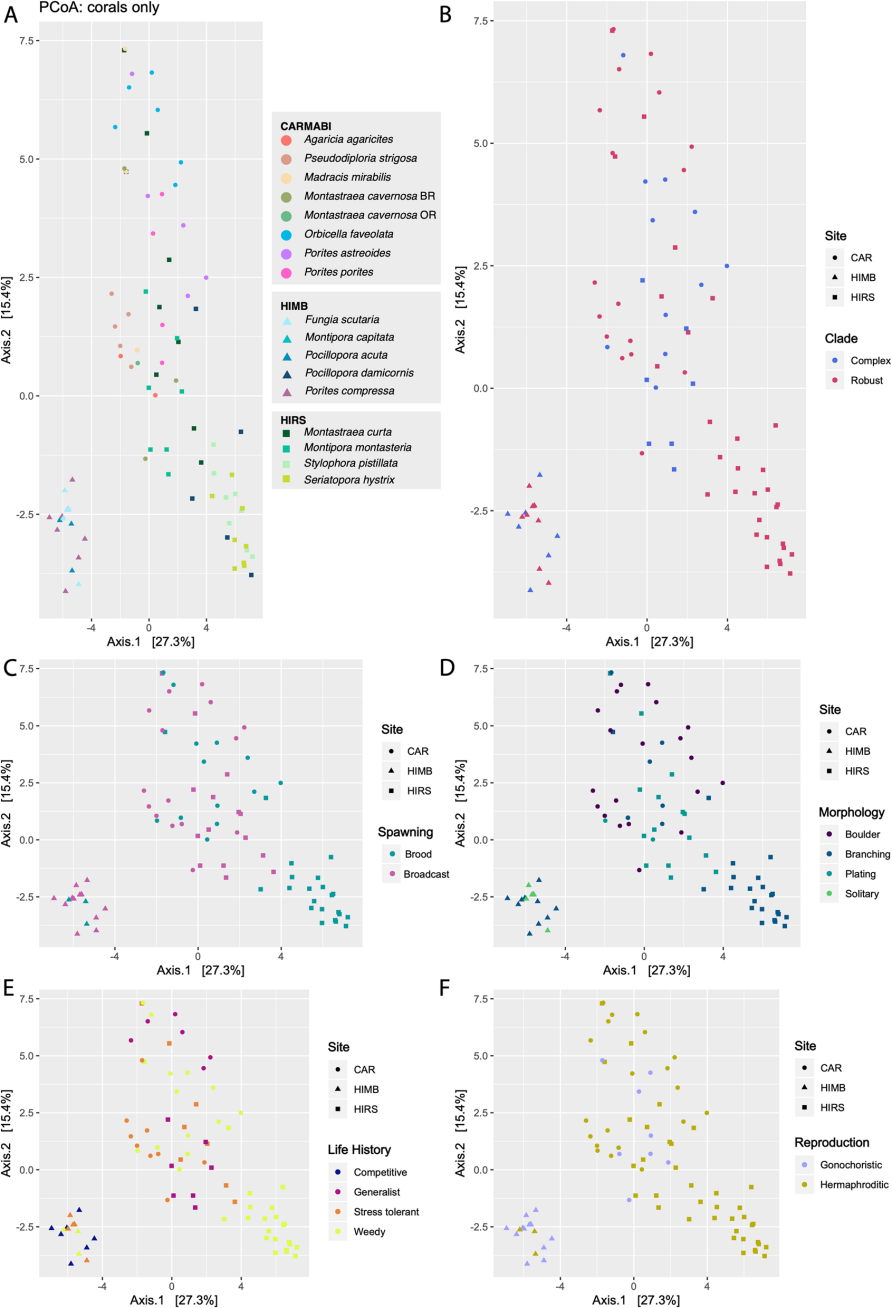
Principal coordinate analysis of *nif*H communities using Euclidean distances on CLR-transformed read counts. Data points are labelled according to **A** host species, **B** clade, **C** reproductive strategy, **D** morphology, **E** life history characteristics, and **F** spawning mode

With respect to beta diversity, the *nif*H composition was significantly different among coral species (Kruskal-Wallis 𝛸^2^_(15)_ = 57, *P* << 0.001) (Fig. 5A), but was not significantly affected by site, reproductive strategy, spawning mode, morphology, ecological strategy, or coral clade (Fig. 5B–F). Specifically, significant differences were detected between *Seriatopora hystrix* and *Porites compressa* (Wilcoxon, adjusted *P* = 0.047) and *S. hystrix* and *Pocillopora acuta* (Wilcoxon, adjusted *P* = 0.047), with both *Pocillopora* species harboring a higher relative abundance of *Rhizobiales nif*H phylotypes and lower abundances of phylotypes associated with *Burkholderiales*, *Desulfovibrionales*, and *Rhodobacterales* compared to *S. hystrix*. And based on 16S rRNA gene sequences, most corals, and seawater samples from each site, contained representatives from six families in the order *Rhizobiales* (Supplemental Fig. 5).

**Fig. 6:**
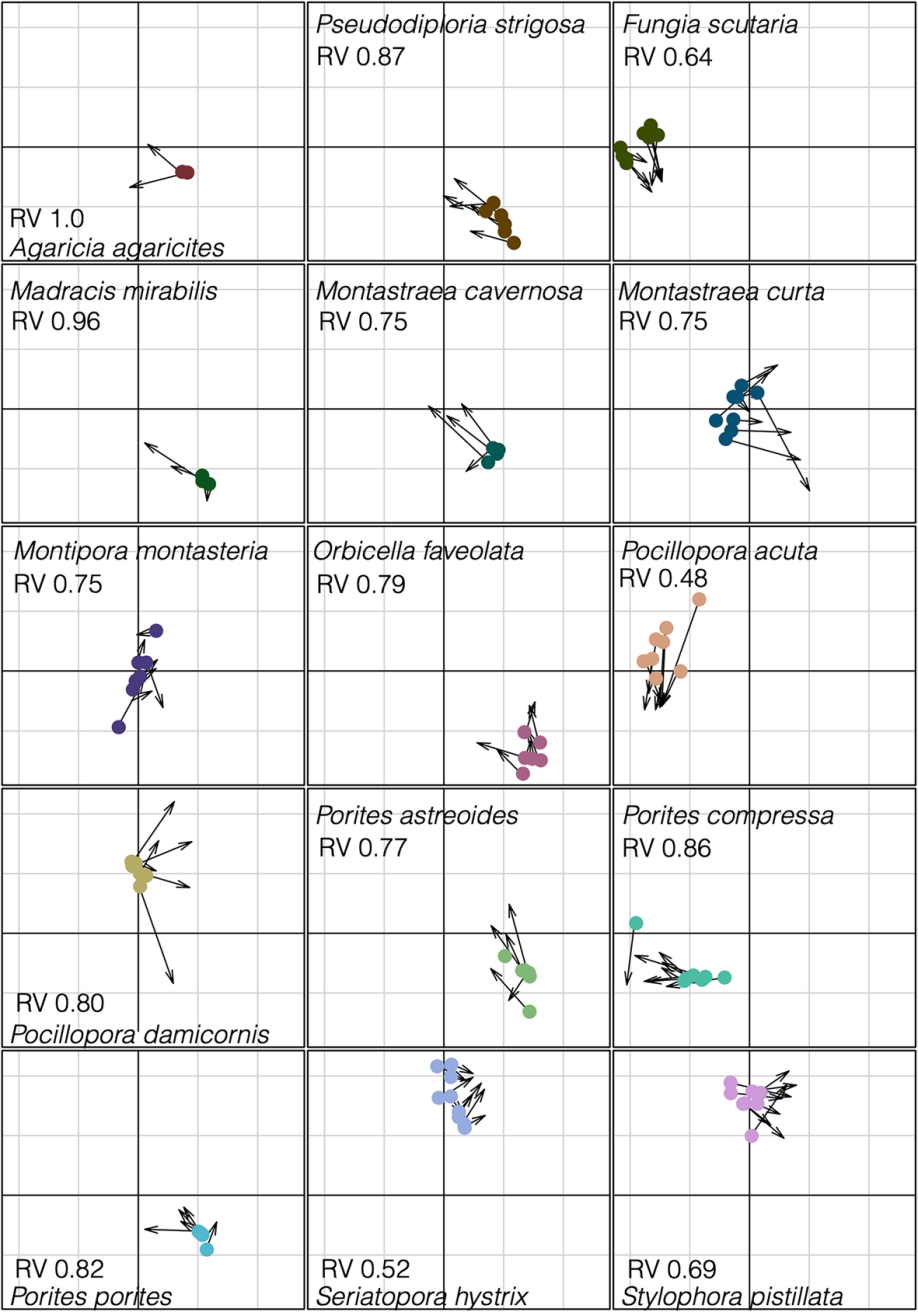
Co-inertia analysis (CIA) for the relationship between *nif*H and 16S rRNA microbial communities. CIA ordinations project 16S rRNA gene (dots) and *nif*H (arrow tips) samples on axes after maximizing covariation among datasets. Length of arrows corresponds to dissimilarity among communities while shared directionality represents positive correlation between 16S rRNA gene and *nif*H relative abundances. RV coefficient (with values 0 to 1) indicating the overall correlation between 16S rRNA and *nif*H gene community compositions, where 0 is no correlation and 1 is maximum correlation

The co-inertia analysis (CIA) was used to test the null hypothesis that 16S rRNA and *nif*H gene communities are highly correlated with each other, given that one is a subset of the other. The analysis revealed that the diazotrophic communities of most coral species are correlated with the rest of the microbiome (Fig. 6). The 16S rRNA and *nif*H gene communities from *Agaricia agaricites*, *Madracis mirabilis*, *Pseudodiploria strigosa*, *Porites compressa*, and *P. porites* were highly correlated (RV coefficients > 0.8), while *Pocillopora acuta* and *Seriatopora hystrix* were the weakly correlated (RV coefficients < 0.6), and the remaining species were moderately correlated (RV coefficients < 0.8 but greater than > 0.6).

**Fig. 7:**
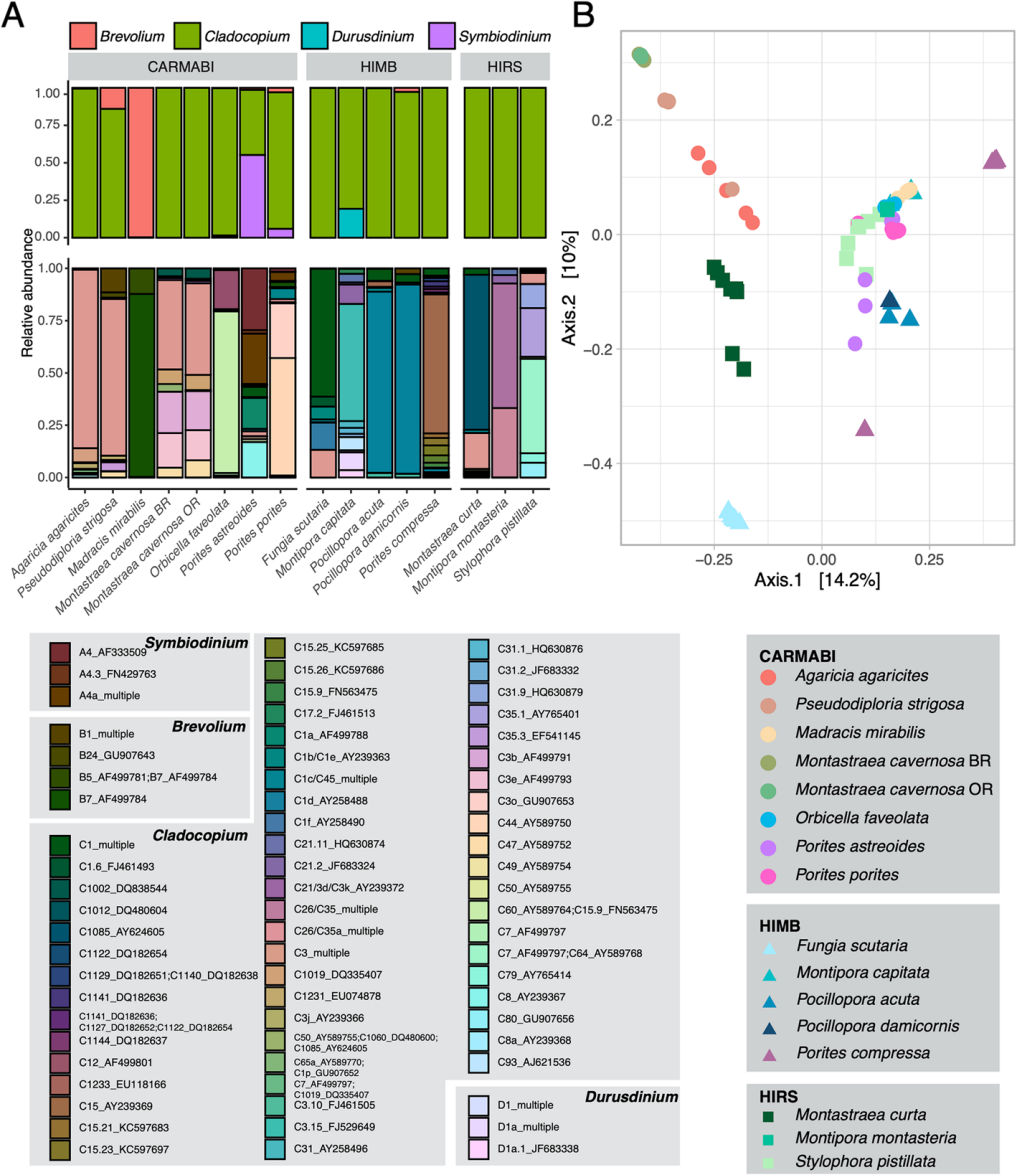
Symbiodiniaceae diversity across coral species including two-color morphologies of *Montastraea cavernosa*, orange (OR) and brown (BR). Average relative abundances for each Symbiodiniaceae genus (**A**) and top-matching NCBI hit for the ITS2 marker across coral species. **B** Principal coordinate ordination of zooxanthellate communities across coral samples using Bray-Curtis distance on rarefied ITS2 counts

Based on ITS2 gene amplicons, Symbiodiniaceae communities varied significantly among coral species in terms of alpha diversity (ANOVA *F*_15,74_ = 9.79, *P* << 0.001). While most coral species were dominated by *Cladocopium* phylotypes (Fig. 7A), *Madracis mirabilis* and *Porites astreoides* harbored higher relative abundances of *Breviolum* and *Symbiodinium* phylotypes, respectively (Fig. 7A). Symbiodiniaceae alpha diversity was also significantly higher among complex corals compared to robust species (nested ANOVA *F*_14,74_ = 5.7, *P* = 0.03), but did not vary by site (nested ANOVA *F*_13,74_ = 0.268, *P* = 0.769), reproductive strategy (nested ANOVA *F*_11,74_ = 0.834, *P* = 0.531), spawning mode (nested ANOVA *F*_14,74_ = 1.82, *P* = 0.199), or ecological strategy (nested ANOVA *F*_12,74_ = 1.14, *P* = 0.369).

Community diversity of Symbiodiniaceae based on beta diversity (Fig. 7 B) was significantly affected by coral species (PERMANOVA *F*_15,74_ = 11.9, *R*^2^ = 0.71, *P* < 0.001) and by sampling site (nested PERMANOVA *F*_2,74_ = 1.79, *P* = 0.009). However, after controlling for species effects, other coral traits and life history characteristics did not significantly impact the beta diversity of Symbiodiniaceae ITS2 ASVs. Coral 16S rRNA communities are not correlated with the Symbiodiniaceae ITS2 communities (Mantel *r* = 0.02, *P* = 0.33).

## Discussion

The microbiome community structure associated with specific coral traits such as reproductive strategy (i.e., gonochoric vs. hermaphroditic), spawning mode (i.e., brooding vs. broadcast spawning), morphology (i.e., boulder vs. branching vs. plating vs. solitary), phylogenetic clade (Superfamilies Complexa vs. Robusta), and ecological life history characteristics (i.e., weedy, competitive, generalist, or stress-tolerant species) were explored using both 16S rRNA gene and *nif*H marker genes on representative scleractinian corals. Based on the 16S rRNA gene analysis, Caribbean coral species had significantly lower alpha diversity (i.e., Shannon diversity index) compared to corals from Hawai’i and Australia. Additionally, alpha diversity was not significantly correlated with reproductive strategy, spawning mode, phylogenetic clade, or ecological characteristics. In comparison, microbiome community composition (i.e., beta diversity) based on the 16S rRNA gene varied between coral species, sites, and ecological life history characteristics but coral morphology had the most significant effect on microbiome communities.

The prokaryotic microbiome community of corals spans the symbiosis continuum from commensal to mutualistic and to parasitic/pathogenic and is dominated by bacteria in the *Gamma*- and *Alphaproteobacteria*, *Actinobacteria*, *Bacteroidetes*, and *Cyanobacteria* [1]. Core members of the coral microbiome include the genus *Endozoicomonas* which was a significant component of the microbiome community for many of the coral species studied here. Functionally, scleractinian corals are often described as nitrogen limited, or dependent on heterotrophic resources for nitrogen [28, 29, 33]. For oligotrophic coral reef ecosystems, the major source of new, versus regenerated, nitrogen is from nitrogen fixation [86], which is mediated primarily by free-living cyanobacteria and heterotrophic bacteria in the oceans [86]. In addition to free-living diazotrophs, several important members of the reef community (i.e., corals and sponges) have the capability to fix nitrogen through symbiotic associations with diazotrophs [32, 87, 88]. It is well-known that the microbiomes of scleractinian corals harbor diazotrophic symbionts [34, 35, 37, 38, 41, 42], as well as other prokaryotes that mediate transformations of inorganic nitrogen in processes such as nitrification, denitrification, and the anaerobic oxidation of ammonium (ANAMMOX) [11, 43, 89–91]. For all corals studied here, members of the bacterial phyla *Chloroflexi*, *Cyanobacteria*, and *Actinobacteria*, known to include diazotrophs, are found either alone or in combination across sites, coral traits, and ecological life history characteristics. Bacterial taxa involved in other steps of the nitrogen cycle (i.e., nitrification and denitrification) are also more common in those coral species that harbor potential diazotrophs. Several of these taxa that can conduct either, or both, nitrification and denitrification include members of the genus *Vibrio*, *Marinobacter*, and *Pseudoaltermonas* [15, 91]. Among the microbiomes of the coral species examined here, members of the genus *Endozoicomonas* varied in abundance with respect to coral species and traits more than any other microbial genus. Although nitrogen fixation has not been reported within this group [92], there is evidence that some *Endozoicomonas* can metabolize DMSP to dimethylsulfide (DMS) [93] which could potentially be catabolized by other microbes to bioavailable sulfur, a co-factor for ferredoxin which is an electron donor to nitrogenase, and in this way could indirectly support nitrogen fixation by diazotrophic members of the microbiome [93].

The phylogenetic placement of *nif*H ASVs from nitrogen-fixing clades suggests that coral diazotroph communities are composed of members from diverse groups with high *nif*H sequence similarity to free-living diazotrophs. These groups include *Proteobacteria* (*Rhizobiales*, *Rhodobacterales*, *Rhodospirillales*, *Burkholderiales*, *Desulfovibrionales*, *Pseudomonadales*, *Chromatiales*) and the photosynthetic green sulfur bacteria *Chlorobiales*. Amplicon sequence variants with similarity to *nif*H sequences from members of the *Firmicutes*, *Cyanobacteria*, and *Actinobacteria* were notably absent in these coral diazotroph communities despite their detection using 16S rRNA gene primers. One potential explanation is that *nif*H primers can be highly variable in their ability to amplify the nitrogenase gene [61] or that not all members of these phyla are diazotrophic.

Several recent studies of coral microbiomes have shown that many coral species harbor non-photosynthetic diazotrophs, often representing the majority of the diazotroph community including some members of the order *Rhizobiales* [35, 38, 41]. In this study, *Rhizobiales* were recovered in 11 of 16 coral species with the highest abundances of *Rhizobiales* found in corals from HIMB, where approximately eight times greater relative abundances were found compared to either CARMABI or HIRS. While not currently considered eutrophic, Kaneohe Bay, HI, where the HIMB samples were collected, has historically been exposed to high amounts of sewage effluent, dredging, and agricultural wastewater [94], which continue to contribute to high concentrations of organic matter in the sediments leading to higher concentrations of NO_x_. It has been suggested [40] that because the *Rhizobiales* are primarily soil bacteria of agricultural importance that the *nif*H sequences recovered from corals identified as belonging to the *Rhizobiales* could be coming from plasmid-borne *nif*H genes horizontally transferred into members of the coral microbiome that could then be exploited by the coral host [95]. However, in this study, *Rhizobiales nif*H sequences were identified in the microbiome of ~75% of the coral species examined from HIMB, while the 16S rRNA gene analysis showed that ~63% of those same coral species harbored *Rhizobiales*. This pattern is not strong support for horizontal gene transfer of plasmid-borne *Rhizobiales nif*H genes. Also, if horizontal gene transfer was occurring, at least in the case of the *Rhizobiales*, we might expect to see a much smaller percentage of taxa identified as *Rhizobiales* based on the 16S rRNA gene data compared to the *nif*H sequence data. However, given the number of species examined (*n* = 16), the sample sizes (CARMABI *n* = 30, HIMB *n* = 23, HIRS *n* = 37), and the multiple sites examined, *Rhizobiales* should be considered a significant component of the microbiome [96]. Supporting a core designation for *Rhizobiales* is the increasing evidence that diazotrophy contributes important amounts of new nitrogen for the coral holobiont under varying environmental and physiological conditions [42, 44, 88].

The co-inertia analysis (CIA) shows that site, coral traits, and life history characteristics did not impact the degree of correlation between the 16S rRNA gene and *nif*H communities for the corals examined in this study. However, despite their lower diazotrophic diversity, the strongest correlation among 16S rRNA and *nif*H communities was for spawning mode where both broadcast spawners (*Pseudodiploria strigosa*, *Porites compressa*) and brooders (*Agaricia agaricites*, *Madracis mirabilis*, and *P. porites*) exhibited highly significant correlations between their 16S rRNA gene and *nif*H communities. The brooders *Pocillopora acuta* and *Seriatopora hystrix*, both branching species, were exceptions to this pattern. These brooding species also demonstrate low overall microbial species richness and are known to represent both weedy and stress-tolerant ecological life history characteristics. They exhibit both resistance to disease and rapid recovery from coral bleaching, which could be attributed to the physiological attributes of their prokaryotic symbionts [3, 97, 98].

Most of the fixed nitrogen in the coral holobiont is found in the Symbiodiniaceae [40–42], and the proportion of Symbiodiniaceae nitrogen demand provided by diazotrophy can be as high as ~15% [42, 44]. Most of the coral species examined here, regardless of location, are dominated by Symbiodiniaceae in the diverse genus *Cladocopium*, which harbors species that are both sensitive and resilient to thermal and light stress. The exceptions are both brooders: *M. mirabilis*, which is dominated by the genus *Breviolum*, known to be a temperature-sensitive member of Symbiodiniaceae [99, 100], and *P. astreiodes* which harbor primarily Symbiodinaceae from the genus *Symbiodinium* many of which are known to be temperature resistant [101]. Additionally, many of the coral species examined here harbor bacteria in the order *Burkholderiales*, which contains the genus *Ralstonia*, a previously described endosymbiont of Symbiodiniaceae that can fix nitrogen [27]. The corals studied here, from both the Caribbean and Pacific, exhibit differences in their functional diazotrophic community based on *nif*H clusters I and III that varies significantly with coral species and sampling site. From a reproductive biology perspective, the corals in this study did show a greater abundance of *Rhizobiales* in gonochoristic corals, but brooders showed highly variable interdependencies between their 16S rRNA and *nif*H communities such that reproductive strategy or spawning mode are likely weak drivers of the diversity, composition, and abundance of coral diazotrophic communities.

As stated above, host species explains the largest amount of the variability in diazotrophic communities presented here. Additionally, local host identity and morphology are the most important drivers of structuring coral host-microbial communities [25], including those taxa that putatively contain diazotrophs. While coral prokaryotic microbiomes can change their community structure in response to local environmental differences [3, 101], here those effects were largely eliminated by collecting corals from similar physical and biochemical environments. This is reflected in the significant, but biologically inconsequential, effects due to site in the analysis of diazotrophic communities. Morphology is a well-known trait that influences both functional and evolutionary trajectories in scleractinian corals [102, 103] and has recently been described as a “supertrait” [104]. Morphology has also been previously suggested as a host trait that is associated with differences in prokaryotic microbiome communities [105, 106], and the partitioning of these communities among species has been shown to be significantly associated with both morphology and the evolutionary history of corals [19]. Here, morphology, when the effect of species is controlled, significantly affects the microbiome communities of corals. Multiple 16S rRNA ASVs were enriched in corals (e.g., *Endozoicomonas*) based on morphology where branching, plating, and solitary corals all had significantly more ASVs with greater differential abundance than those found in boulder morphologies. For diazotrophs, however, only host species significantly affected their abundance, while no other trait, including morphology, was identified as a significant influence on diazotroph abundance.

## Conclusions

For scleractinian corals, from widely separated Caribbean and Pacific locations that varied little in their environmental characteristics such as temperature and irradiance, the prokaryotic microbiome community composition based on the 16S rRNA gene varied as a function of coral species and sites, coral morphology, and ecological life history strategy. For most coral species, their microbiome communities were significantly associated with their diazotrophic components based on a co-inertia analysis of 16S rRNA and *nif*H gene sequences. But while diazotrophs are common and important members of the coral microbiome, only host species differences influenced the diazotrophic community in the corals in this study. *Rhizobiales*, a common diazotroph, were identified using both 16S rRNA and *nif*H gene sequences from seawater, sediment porewater, and corals. Morphology, an important evolutionary trait of scleractinian corals, was the strongest source of variation for the microbiome of the corals studied here while diazotrophic communities varied solely based on host species. Morphology, or the shape and size of corals, affects many important physiological attributes such as the capture of light and particulate food, flow modulated gas exchange, and the exchange of metabolites (e.g., nutrients) between the environment and the coral, as well as within the multicompartmental structure (i.e., skeleton, tissues, and mucous) of the coral host. These processes all influence important ecological outcomes for corals on reefs. Many of these processes, especially nutrient cycling, are mediated to a large extent by the unique physiological signature of the coral microbiome. To better understand the multiple roles of the coral microbiome in host health and disease, identifying and understanding the evolutionary drivers (e.g., morphology) of microbiome character states is essential in order to quantify the effects of human perturbation on coral reefs.

## Supplementary Information

**Additional file 1.** Supplementary material containing Tables S1–S3 and Figures S1–S5.

## Data Availability

Raw 16S rRNA, *nifH*, and Symbiodiniaceae ITS2 reads have been submitted to the NCBI Sequence Read Archive under BioProject accession number PRJNA498285. All other data will be made available by contacting the corresponding author.
