# Supplementary material for: Community structure of coral microbiomes is dependent on host morphology
Source: Microbiome. 2022 Jul 28;10:113. doi: 10.1186/s40168-022-01308-w (PMC9331152; doi:10.1186/s40168-022-01308-w)
Supplement: Supplementary file 2 — Additional file 1. Supplementary material containing Tables S1–S3 and Figures S1–S5. [file 40168_2022_1308_MOESM1_ESM.pdf]

SUPPLEMENTAL MATERIAL

Community Structure of Coral Microbiomes Are Dependent on Host Morphology

Kathleen M. Morrow[1,3], Sabrina M. Pankey[1], Michael P. Lesser[1,2]*

[1]University of New Hampshire, Department of Molecular, Cellular and Biomedical Sciences, Durham, NH 03824, USA

[2]University of New Hampshire, School of Marine Science and Ocean Engineering, Durham, NH 03824, USA

[3]Current Address: Thomas Jefferson High School for Science and Technology, 6560 Braddock Rd., Alexandria, VA 22312, USA

*Corresponding author; Email: mpl@unh.edu

Keywords: Coral, 16S rRNA, diazotroph, microbiome, nitrogen fixation, Symbiodiniaceae, *nif*H

Running title: Diazotrophic communities in corals

Table S1. Collection sites and ecological trait data for coral species. CAR= Curaçao (CARMABI), HIMB=Hawaii (HIMB), HIRS=Australia (HIRS), BR=brown, OR=orange, SM=spawning mode, RS=reproductive strategy; H=hermaphroditic, G=Gonochoristic. * seasonal daytime spawning of sperm only has been observed in individuals from HIMB while this species releases planulae year round (Schmidt-Roach 2014)

Species	*Site*	*Ecology*	*Morphology*	*Clade*	*SM*	*RS*
Agaricia agaricites	CAR	Weedy	Plating	Complex	brood	H
Pseudodiploria strigosa	CAR	Stress tolerant	Boulder	Robust	spawn	H
Fungia scutaria	HIMB	Stress tolerant	Solitary	Robust	spawn	G
Madracis mirabilis	CAR	Weedy	Branching	Robust	brood	H
Montastraea cavernosa BR	CAR	Stress tolerant	Boulder	Robust	spawn	G
Montastraea cavernosa OR	CAR	Stress tolerant	Boulder	Robust	spawn	G
Montastraea curta	HIRS	Stress tolerant	Plating	Robust	spawn	H
Montipora monasteriata	HIRS	Generalist	Plating	Complex	spawn	H
Montipora capitata	HIMB	Competitive	Branch/Plate	Complex	spawn	H
Orbicella faveolata	CAR	Generalist	Boulder	Robust	spawn	H
Pocillopora acuta	HIMB	Weedy	Branching	Robust	brood*	H
Pocillopora damicornis	HIRS	Weedy	Branching	Robust	brood	H
Porites astreoides	CAR	Weedy	Boulder	Complex	brood	H
Porites compressa	HIMB	Competitive	Branching	Complex	spawn	G
Porites porites	CAR	Weedy	Branching	Complex	brood	G
Seriatopora hystrix	HIRS	Weedy	Branching	Robust	brood	H
Stylophora pistillata	HIRS	Weedy	Branching	Robust	brood	H

Schmidt-Roach S, Johnston E, Fontana S, Jury CP, Forsman Z.2014. Daytime spawning of Pocillopora acuta in Kaneohe Bay, Hawai'i. Galaxea 16: 11-12.

Table S2. Library sizes and NCBI Short Read Archive accessions for 16S, nifH and ITS2 amplicons. Library sizes shown before and after filtering for both quality via DADA2 and contaminants (e.g. chloroplasts, mitochondria, single ASV's) via PhyloSeq in R.

				16S amplicon library			nifH amplicon library			ITS2 amplicon library		
	Species	library ID	BioSample	SRA accession	read counts (merged)	filtered counts	SRA accession	read counts (merged)	filtered counts	SRA accession	read counts (merged)	filtered counts
Curaçao CARMABI	*Agaricia agaricites*	AA1_CAR	SAMN10286531	SRR8105310	37834	3915	SRR8104648	56712	9797	SRR15292589	52285	39029
		AA2_CAR	SAMN10286532	SRR8105315	49141	5403	SRR8104651	68290	0			
		AA3_CAR	SAMN10286533	SRR8105316	65072	7528	SRR8104650	45809	0	SRR15292588	55238	38153
		AA4_CAR	SAMN10286534	SRR8105179	33472	4868	SRR8104645	58963	0	SRR15292552	44899	29284
		AA5_CAR	SAMN10286535	SRR8105251	51532	6259	SRR8104654	58770	0	SRR15292541	43090	30706
		AA6_CAR	SAMN10286536	SRR8105250	64464	8008	SRR8104655	59719	4258	SRR15292530	43666	33269
	Pseudodiploria strigosa	DS1_CAR	SAMN10286537	SRR8105254	37464	2405	SRR8104641	54399	30023	SRR15292519	43947	34131
		DS2_CAR	SAMN10286538	SRR8105313	48747	3594	SRR8104638	57618	3608			
		DS3_CAR	SAMN10286539	SRR8105307	13517	0	SRR8104659	17279	0	SRR15292508	52341	32616
		DS4_CAR	SAMN10286540	SRR8105311	50962	2190	SRR8104643	75596	36552	SRR15292497	45229	33431
		DS5_CAR	SAMN10286541	SRR8105312	71131	4199	SRR8104642	57054	27484	SRR15292486	41128	31164
		DS6_CAR	SAMN10286542	SRR8105309	35460	2468	SRR8104649	61677	30980	SRR15292475	48517	36596
	Madracis mirabilis	Mad1_CAR	SAMN10286543	SRR8105257	57231	3660	SRR8104630	50996	0	SRR15292553	44040	31213
		Mad2_CAR	SAMN10286544	SRR8105256	54013	4229	SRR8104629	89616	0	SRR15292551	41506	29907
		Mad3_CAR	SAMN10286545	SRR8105213	56806	3759	SRR8104628	50841	0	SRR15292550	37171	27519
		Mad4_CAR	SAMN10286546	SRR8105207	69294	5869	SRR8104601	55845	1609	SRR15292549	39371	28922
		Mad5_CAR	SAMN10286547	SRR8105206	55092	3195	SRR8104639	40238	509	SRR15292548	40404	29079
		Mad6_CAR	SAMN10286548	SRR8105209	52104	3310	SRR8104636	54342	1355	SRR15292547	44770	31819
	Montastraea cavernosa	MCBR1_CAR	SAMN10286549	SRR8105208	62391	5817	SRR8104637	55368	0	SRR15292525	52447	38641
		MCBR2_CAR	SAMN10286550	SRR8105276	61369	4963	SRR8104634	55868	0	SRR15292524	45452	34218
		MCBR3_CAR	SAMN10286551	SRR8105277	53140	3224	SRR8104635	56755	977	SRR15292523	56942	41519
		MCBR4_CAR	SAMN10286552	SRR8105272	53542	5100	SRR8104623	52519	520	SRR15292522	45178	32019
		MCBR5_CAR	SAMN10286553	SRR8105228	71089	8605	SRR8104622	54895	2567	SRR15292521	45886	35740
		MCBR6_CAR	SAMN10286554	SRR8105215	42741	3788	SRR8104627	59195	0	SRR15292520	46229	32669
		MCOR1_CAR	SAMN10286555	SRR8105214	45439	2805	SRR8104656	55179	0	SRR15292518	44746	34118
		MCOR2_CAR	SAMN10286556	SRR8105211	55368	2951	SRR8104657	58416	0	SRR15292517	43062	33336
		MCOR3_CAR	SAMN10286557	SRR8105210	69113	3865	SRR8104658	56808	1440	SRR15292516	46943	34542
	Orbicella faveolata	OF1_CAR	SAMN10286568	SRR8105249	47989	4255	SRR8104602	65021	1828	SRR15292505	41132	25073
		OF2_CAR	SAMN10286569	SRR8105248	59342	3852	SRR8104597	53666	864	SRR15292504	45942	25847
		OF3_CAR	SAMN10286570	SRR8105255	52985	4932	SRR8104613	58815	2167	SRR15292503	44934	28299
		OF4_CAR	SAMN10286571	SRR8105253	37279	2761	SRR8104620	67796	1226	SRR15292502	40782	25366
		OF5_CAR	SAMN10286572	SRR8105252	43403	2391	SRR8104621	59937	687	SRR15292501	47919	26441
		OF6_CAR	SAMN10286573	SRR8105306	41364	2641	SRR8104618	56609	2647	SRR15292500	43617	25866
	Porites astreoides	PA1_CAR	SAMN10286574	SRR8105181	59271	5483	SRR8104644	59239	1081			
		PA2_CAR	SAMN10286575	SRR8105164	52675	6586	SRR8104647	54262	2611	SRR15292499	57880	36583
		PA3_CAR	SAMN10286576	SRR8105233	38967	3893	SRR8104646	57903	2532	SRR15292498	53664	37643
		PA4_CAR	SAMN10286577	SRR8105230	41012	4085	SRR8104632	45883	610	SRR15292496	61714	37717
		PA5_CAR	SAMN10286578	SRR8105212	34473	3071	SRR8104633	58992	0	SRR15292495	50273	31555
		PA6_CAR	SAMN10286579	SRR8105268	49888	4987	SRR8104640	54635	765	SRR15292494	43756	29325
	Porites porites	PP1_CAR	SAMN10286580	SRR8105273	72742	8767	SRR8104619	49989	1210	SRR15292468	46346	35354
		PP2_CAR	SAMN10286581	SRR8105274	69107	7907	SRR8104616	61188	2801	SRR15292467	43487	34351
		PP3_CAR	SAMN10286582	SRR8105275	75736	9148	SRR8104626	45744	753	SRR15292466	51997	35437
		PP4_CAR	SAMN10286583	SRR8105269	73820	9740	SRR8104625	57170	1963	SRR15292465	54753	38998
		PP5_CAR	SAMN10286584	SRR8105270	87816	13456	SRR8104624	57812	637	SRR15292586	51461	37256
		PP6_CAR	SAMN10286585	SRR8105271	74905	9864	SRR8104631	53149	0	SRR15292585	50211	36263
		PW1_CAR	SAMN10286558	SRR8105234	69038	0	SRR8104662	63437	606			
	Seawater	SW1_CAR	SAMN10286563	SRR8105162	60807	3913	SRR8104669	114306	0			
		SW2_CAR	SAMN10286564	SRR8105161	49176	4627	SRR8104668	89011	0			
		SW3_CAR	SAMN10286565	SRR8105160	69515	5469	SRR8104667	92628	0			
		SW4_CAR	SAMN10286566	SRR8105235	68716	5020	SRR8104666	136209	0			
		SW5_CAR	SAMN10286567	SRR8105236	55713	4736	SRR8104665	149488	0			
	Sediment (Porewater)	PW2_CAR	SAMN10286559	SRR8105231	68227	0	SRR8104660	64622	0			
		PW3_CAR	SAMN10286560	SRR8105232	58145	0	SRR8104661	77361	0			
		PW4_CAR	SAMN10286561	SRR8105229	51871	0	SRR8104652	100729	0			
		PW5_CAR	SAMN10286562	SRR8105227	50261	0	SRR8104653	89643	0			
	Fungia scutaria	FS1_HIMB	SAMN10286586	SRR8105223	64199	18343	SRR8104704	40664	3118	SRR15292587	41747	29976
		FS2_HIMB	SAMN10286587	SRR8105224	59905	18086	SRR8104705	65687	21488	SRR15292576	41864	30624
		FS3_HIMB	SAMN10286588	SRR8105217	57856	19511	SRR8104690	23111	9996	SRR15292565	44656	32719
		FS4_HIMB	SAMN10286589	SRR8105218	55075	18231	SRR8104689	54690	6934	SRR15292559	35779	25601
		FS5_HIMB	SAMN10286590	SRR8105219	62503	22079	SRR8104688	53727	29795	SRR15292558	40254	29159
		FS6_HIMB	SAMN10286591	SRR8105220	58360	20748	SRR8104687	55332	2380	SRR15292557	32110	23456
		FS7_HIMB	SAMN10286592	SRR8105225	59423	18784				SRR15292556	28549	20297
		FS8_HIMB	SAMN10286593	SRR8105226	56914	14765				SRR15292555	36760	26713
		FS9_HIMB	SAMN10286594	SRR8105201	63838	20099	SRR8104686	53858	25905	SRR15292554	35089	24187
	Montipora capitata	MC10c_HIMB	SAMN10286595	SRR8105184	80933	2713						
		MC11c_HIMB	SAMN10286596	SRR8105185	79218	6014				SRR15292544	44799	32229
		MC12c_HIMB	SAMN10286597	SRR8105159	72450	6981				SRR15292543	37815	27232
		MC13c_HIMB	SAMN10286598	SRR8105158	72202	3415				SRR15292542	30198	21678
		MC14c_HIMB	SAMN10286599	SRR8105180	82008	8639				SRR15292540	37776	25085
		MC4s_HIMB	SAMN10286600	SRR8105167	75389	7891	SRR8104691	25628	0	SRR15292537	33884	0
		MC5s_HIMB	SAMN10286601	SRR8105166	74557	6145	SRR8104615	31843	0	SRR15292535	30026	0
		MC6s_HIMB	SAMN10286602	SRR8105165	69367	5450	SRR8104614	41331	0	SRR15292533	32524	0
		MC7s_HIMB	SAMN10286603	SRR8105163	47402	0	SRR8104617	71557	0	SRR15292531	32304	0
		MC8c_HIMB	SAMN10286604	SRR8105182	65656	0				SRR15292528	37242	0
		MC9c_HIMB	SAMN10286605	SRR8105183	83739	8141				SRR15292526	34909	24779

Table S2. (continued)

Hawai'i HIMB	*Pocillopora acuta*	PD1_HIMB	SAMN10286626	SRR8105176	55204	13111	SRR8104664	58319	5435			
		PD2_HIMB	SAMN10286627	SRR8105173	67109	15091	SRR8104663	68362	10375			
		PD3_HIMB	SAMN10286628	SRR8105174	61945	15109	SRR8104706	25402	1313			
		PD4_HIMB	SAMN10286629	SRR8105171	74100	16522	SRR8104707	88719	2033			
		PD5_HIMB	SAMN10286630	SRR8105172	49736	11559	SRR8104708	58772	6212	SRR15292473	37499	33498
		PD6_HIMB	SAMN10286631	SRR8105169	35058	11547	SRR8104709	36691	14443	SRR15292472	23607	27624
		PD7_HIMB	SAMN10286632	SRR8105151	59460	16027	SRR8104710	50538	1502	SRR15292471	46979	17476
		PD8_HIMB	SAMN10286633	SRR8105150	53933	15343	SRR8104711	57434	4161	SRR15292470	35273	31964
	Porites compressa	PC1_HIMB	SAMN10286634	SRR8105153	56745	16052	SRR8104671	69061	5237	SRR15292493	33442	22424
		PC2_HIMB	SAMN10286635	SRR8105152	94353	29522	SRR8104670	57188	11948	SRR15292492	40179	27833
		PC3_HIMB	SAMN10286636	SRR8105147	96364	29163	SRR8104685	40069	12178	SRR15292491	50242	33488
		PC4_HIMB	SAMN10286637	SRR8105146	82227	24817	SRR8104684	38078	5158	SRR15292490	35918	25230
		PC5_HIMB	SAMN10286638	SRR8105149	62281	19454	SRR8104683	50052	25873	SRR15292489	42440	28010
		PC6_HIMB	SAMN10286639	SRR8105148	51387	16625	SRR8104682	55396	34411	SRR15292488	43334	29224
		PC7_HIMB	SAMN10286640	SRR8105144	70549	22150				SRR15292487	30554	20917
		PC8_HIMB	SAMN10286641	SRR8105143	58974	15167	SRR8104702	57722	2558	SRR15292485	37458	25150
		PC9_HIMB	SAMN10286642	SRR8105222	56960	16646	SRR8104703	57740	13177	SRR15292484	39758	27489
	Seawater	SW1_HIMB	SAMN10286616	SRR8105194	72676	21445						
		SW10_HIMB	SAMN10286617	SRR8105290	81392	25709						
		SW2_HIMB	SAMN10286618	SRR8105288	8684	0						
		SW3_HIMB	SAMN10286619	SRR8105287	68893	20303						
		SW4_HIMB	SAMN10286620	SRR8105285	64278	19902						
		SW5_HIMB	SAMN10286621	SRR8105284	58093	19589						
		SW6_HIMB	SAMN10286622	SRR8105283	64919	20048						
		SW7_HIMB	SAMN10286623	SRR8105282	2981	0						
		SW8_HIMB	SAMN10286624	SRR8105281	65800	22058						
		SW9_HIMB	SAMN10286625	SRR8105291	69833	23031						
	Sediment (Porewater)	PW1_HIMB	SAMN10286606	SRR8105292	85752	15939						
		PW10_HIMB	SAMN10286607	SRR8105195	83816	17464						
		PW2_HIMB	SAMN10286608	SRR8105293	65028	13033						
		PW3_HIMB	SAMN10286609	SRR8105314	73462	16312						
		PW4_HIMB	SAMN10286610	SRR8105193	69740	17133						
		PW5_HIMB	SAMN10286611	SRR8105192	69545	14414						
		PW6_HIMB	SAMN10286612	SRR8105187	61794	14726						
		PW7_HIMB	SAMN10286613	SRR8105186	60339	13784						
		PW8_HIMB	SAMN10286614	SRR8105189	77282	14244						
		PW9_HIMB	SAMN10286615	SRR8105188	86305	17888						
Australia HIRS	*Montastraea curta*	MC1_HIRS	SAMN10286466	SRR8105155	57735	15332	SRR8104676	197028	3567	SRR15292546	33178	26140
		MC10_HIRS	SAMN10286467	SRR8105301	62467	14497	SRR8104713	130636	18946	SRR15292545	25507	0
		MC2_HIRS	SAMN10286468	SRR8105303	66332	16659	SRR8104609	166773	878	SRR15292539	33332	25790
		MC3_HIRS	SAMN10286469	SRR8105145	65702	16322	SRR8104606	194819	21741	SRR15292538	40241	29974
		MC4_HIRS	SAMN10286470	SRR8105142	80543	19128	SRR8104604	159826	641			
		MC5_HIRS	SAMN10286471	SRR8105318	72678	16279	SRR8104678	165084	4208	SRR15292536	37965	28320
		MC6_HIRS	SAMN10286472	SRR8105317	73955	20015	SRR8104672	162366	3350	SRR15292534	35913	27967
		MC7_HIRS	SAMN10286473	SRR8105168	70033	15859	SRR8104698	157322	0	SRR15292532	33802	25571
		MC8_HIRS	SAMN10286474	SRR8105156	100683	23953	SRR8104694	64532	5135	SRR15292529	31803	25480
		MC9_HIRS	SAMN10286475	SRR8105300	74946	18733	SRR8104716	109614	0	SRR15292527	41845	28325
	Montipora monasteriata	MM1_HIRS	SAMN10286481	SRR8105298	85215	19275	SRR15390846	130979	891	SRR15292511	33859	0
		MM2_HIRS	SAMN10286482	SRR8105296	79250	22019	SRR15390845	162252	667	SRR15292510	41136	0
		MM3_HIRS	SAMN10286483	SRR8105297	77006	15970	SRR15390844	56868	0	SRR15292509	38277	0
		MM4_HIRS	SAMN10286484	SRR8105294	74835	19398	SRR15390843	147288	3096	SRR15292507	42351	0
		MM5_HIRS	SAMN10286485	SRR8105295	71478	18401	SRR15390842	164169	696	SRR15292506	44588	0
	Montipora foliosa	MF1_HIRS	SAMN10286476	SRR8105289	70229	19013	SRR15390841	39165	0	SRR15292515	36724	28759
		MF2_HIRS	SAMN10286477	SRR8105216	47260	9198	SRR15390840	98232	0	SRR15292514	41136	31629
		MF3_HIRS	SAMN10286478	SRR8105304	64089	13638	SRR15390839	126475	1252			
		MF4_HIRS	SAMN10286479	SRR8105305	41327	8607	SRR15390838	125872	1568	SRR15292513	35064	0
		MF5_HIRS	SAMN10286480	SRR8105157	48049	7225	SRR15390837	90461	8930	SRR15292512	34068	0
	Pocillopora damicornis	PD10_HIRS	SAMN10286496	SRR8105263	56564	15813	SRR8104595	57055	0	SRR15292483	41847	0
		PD11_HIRS	SAMN10286497	SRR8105266	57388	17766	SRR8104607	89115	1761	SRR15292482	32825	0
		PD12_HIRS	SAMN10286498	SRR8105267	68381	17511	SRR8104699	127238	6272	SRR15292481	35404	0
		PD13_HIRS	SAMN10286499	SRR8105237	59142	17920	SRR8104610	91516	979	SRR15292480	39254	0
		PD14_HIRS	SAMN10286500	SRR8105240	50028	15302	SRR8104674	107909	2369	SRR15292479	37286	0
		PD15_HIRS	SAMN10286501	SRR8105239	47934	15118	SRR8104679	8041	0	SRR15292478	34131	0
		PD16_HIRS	SAMN10286502	SRR8105242	53514	17095				SRR15292477	34978	0
		PD17_HIRS	SAMN10286503	SRR8105241	46606	12612				SRR15292476	39025	0
		PD18_HIRS	SAMN10286504	SRR8105244	58696	14856	SRR8104700	49670	1771	SRR15292474	50897	0
		PD9_HIRS	SAMN10286505	SRR8105262	51963	15213	SRR8104720	96713	19824	SRR15292469	46470	0
	Seriatopora hystrix	SH1_HIRS	SAMN10286506	SRR8105243	57706	19709	SRR8104717	91583	2818	SRR15292584	34324	0
		SH2_HIRS	SAMN10286507	SRR8105246	58583	22303	SRR8104721	74020	3698	SRR15292583	35248	0
		SH3_HIRS	SAMN10286508	SRR8105245	58228	20731				SRR15292582	35725	0
		SH4_HIRS	SAMN10286509	SRR8105308	53491	18357	SRR8104594	54370	1156	SRR15292581	36121	0
		SH4b_HIRS	SAMN10286510	SRR8105264	46718	18971	SRR8104608	25368	958	SRR15292580	42286	0
		SH5_HIRS	SAMN10286511	SRR8105278	42170	12793	SRR8104600	71896	2194	SRR15292579	38133	0
		SH5b_HIRS	SAMN10286512	SRR8105265	41355	9414	SRR8104675	139201	9312	SRR15292578	48204	0
		SH6_HIRS	SAMN10286513	SRR8105279	43728	15898	SRR8104701	113561	1950	SRR15292577	41248	0
		SH7_HIRS	SAMN10286514	SRR8105280	42972	10342	SRR8104611	90964	7076	SRR15292575	44706	0
		SH8_HIRS	SAMN10286515	SRR8105247	67429	19503				SRR15292574	38808	29701
	Stylophora pistillata	SP1a_HIRS	SAMN10286516	SRR8105286	45188	14452				SRR15292573	45248	0
		SP1b_HIRS	SAMN10286517	SRR8105221	46946	14558	SRR8104596	70285	1714			
		SP1c_HIRS	SAMN10286518	SRR8105203	31207	10621	SRR8104612	198730	1428			
		SP2a_HIRS	SAMN10286519	SRR8105177	37478	10080				SRR15292572	36139	24867

		SP2b_HIRS	SAMN10286520	SRR8105200	29705	9985				SRR15292571	39430	27890
		SP2c_HIRS	SAMN10286521	SRR8105202	35934	12491	SRR8104593	152576	962	SRR15292570	38752	0
		SP3a_HIRS	SAMN10286522	SRR8105178	37213	13671				SRR15292569	41534	0
		SP3b_HIRS	SAMN10286523	SRR8105199	55409	18217	SRR8104599	77336	0	SRR15292568	44962	0
		SP3c_HIRS	SAMN10286524	SRR8105197	36150	10836	SRR8104692	117347	1788	SRR15292567	29291	0
		SP4a_HIRS	SAMN10286525	SRR8105175	40149	14026	SRR8104697	5892	0	SRR15292566	37132	26534
		SP4b_HIRS	SAMN10286526	SRR8105198	37608	14003	SRR8104715	105536	1109	SRR15292564	36681	24672
		SP4c_HIRS	SAMN10286527	SRR8105196	37191	13482	SRR8104718	120320	2227	SRR15292563	37643	27470
		SP5a_HIRS	SAMN10286528	SRR8105170	40631	10172	SRR8104680	128007	1641	SRR15292562	52531	26205
		SP5b_HIRS	SAMN10286529	SRR8105204	58887	18989				SRR15292561	35137	23574
		SP5c_HIRS	SAMN10286530	SRR8105260	37715	13049	SRR8104714	50687	1214	SRR15292560	37389	27869
	Seawater	SW1_HIRS	SAMN10286491	SRR8105191	63775	20028	SRR8104719	69944	799			
		SW2_HIRS	SAMN10286492	SRR8105205	61832	20314						
		SW3_HIRS	SAMN10286493	SRR8105261	66542	20671	SRR8104598	98501	0			
		SW4_HIRS	SAMN10286494	SRR8105258	59549	19706	SRR8104605	57055	0			
		SW5_HIRS	SAMN10286495	SRR8105259	60920	20043	SRR8104603	68174	0			
	Sediment (Porewater)	PW1_HIRS	SAMN10286486	SRR8105238	62148	9548	SRR8104712	70133	1413			
		PW2_HIRS	SAMN10286487	SRR8105302	60620	11335	SRR8104677	87801	734			
		PW3_HIRS	SAMN10286488	SRR8105154	71579	14054	SRR8104681	105171	892			
		PW4_HIRS	SAMN10286489	SRR8105299	54638	11039	SRR8104696	112439	2872			
		PW5_HIRS	SAMN10286490	SRR8105190	62523	10036	SRR8104695	115330	2150			

Table S3. Summary of numbers of bacteria differentially enriched in 16S rRNA sequences with respect to coral species, sampling site, and life history traits. Significant ASVs determined through Wald tests via DESeq2. Coral traits: Morphology (mounding, branching, plating, solitary), Reproductive Strategy (RS; gonochoristic or hermaphroditic), Spawning mode (SM; brooding, spawning), Ecological life history charateristics (weedy, competitive, generalist, stress-tolerant), Phylogenetic clade (robust, complex).

Microbial group	*Microbial Genus*	*Species*	*Site*	*Morph*	*RS*	*SM*	*Ecology*	*Clade*
Acidobacteria	unassigned	1		8			3	
Actinobacteria	unassigned			1		1	1	
Alphaproteobacteria	*Bradyrhizobium*		1	1				
	Defluviicoccus			1			1	
	Holospora			1				
	Pseudahrensia			1				
	Rhodobium		1	1				
	Rickettsia			1				
	Ruegeria	2	5	3			1	
	Shimia	2	4	7				
	Thalassospira	9	11	14			2	4
	unassigned	3	15	16		1	3	
Bacteriodetes	*Actibacter*						1	
	Fabibacter						1	
	Maritimimonas	3	6	4			2	
	Prolixibacter		1	1			1	1
	Winogradskyella		1	1				
	unassigned			3				
Betaproteobacteria	*Bordetella*			1				
	Branchiomonas	7	10	12			6	2
	Delftia		10			9	8	9
	Ralstonia		1					
	unassigned							

Caldilineae	unassigned							
Chlorobi	*Prosthecochloris*		1	2				
Chloroflexi	unassigned		2	3			1	
Cyanobacteria	*Synechococcus*	3	10	2			1	
Cyanobacteria	unassigned		1	1			1	
Cytophagia	*Ameobophilus*							
Deltaproteobacteria	unassigned		2	5				1
Firmicutes	*Staphylococcus*			1				
Fusobacteria	*Propionigenium*	1	1	2			1	
Gammaproteobacteria	*Acinetobacter*		2					
	Aestuariibacter			1				
	Alteromonas	10		1			1	
	Endozoicomonas	22	55	44	7	15	29	26
	Haliea			1				
	Marine Methylotrophic Group 3	1						
	Nitrosococcus			1				
	Photobacterium	2	3	3				1
	Pseudoalteromonas	3	9	14			13	1
	Vibrio	13	23	19				2
	unassigned			3				
Gemmatimondetes	unassigned		1	3			1	
Nitrospirae	*Nitrospira*	1		4				
Tenericutes	unassigned	1	1	1				
	Nitrosopumilus	6	8	2				
	unassigned	1	5	5			1	
Verrucomicrobia	*Rubritalea*		1	2				
**unassigned**	unassigned	4	66	64	21	15	34	19
	**Total ASVs**	**95**	**257**	**261**	**28**	**41**	**113**	**66**

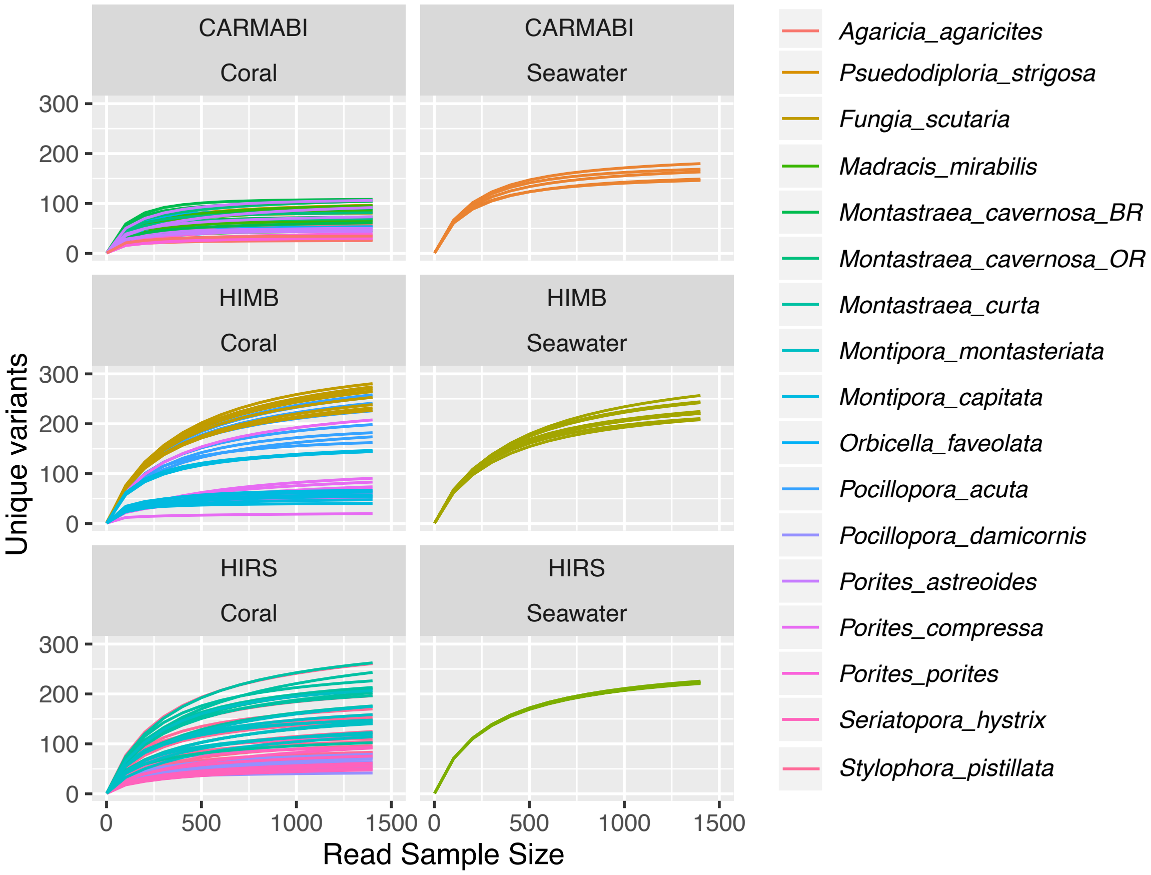

Figure S1. Rarefaction curves for 16S rRNA read libraries for all seawater and coral samples.

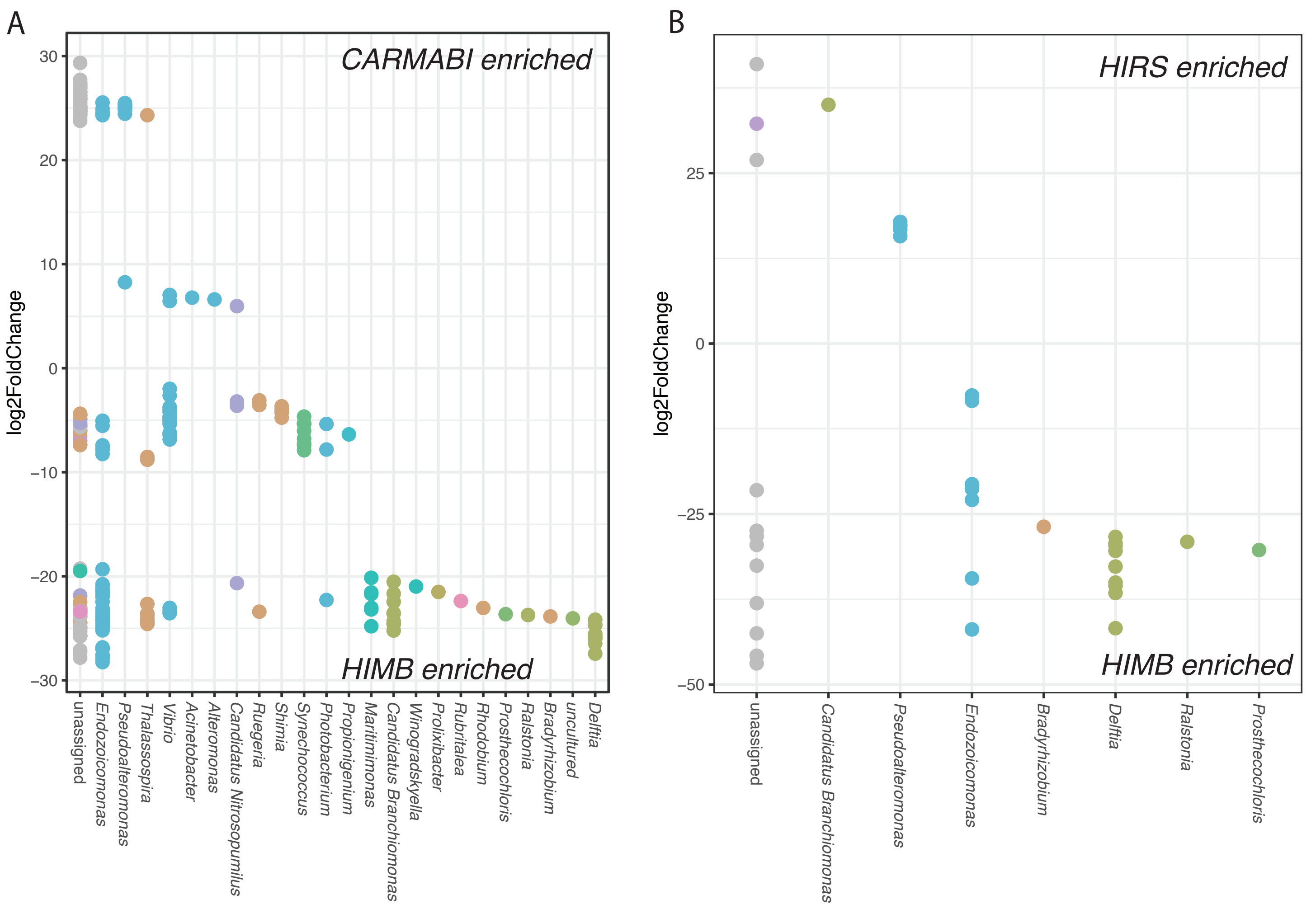

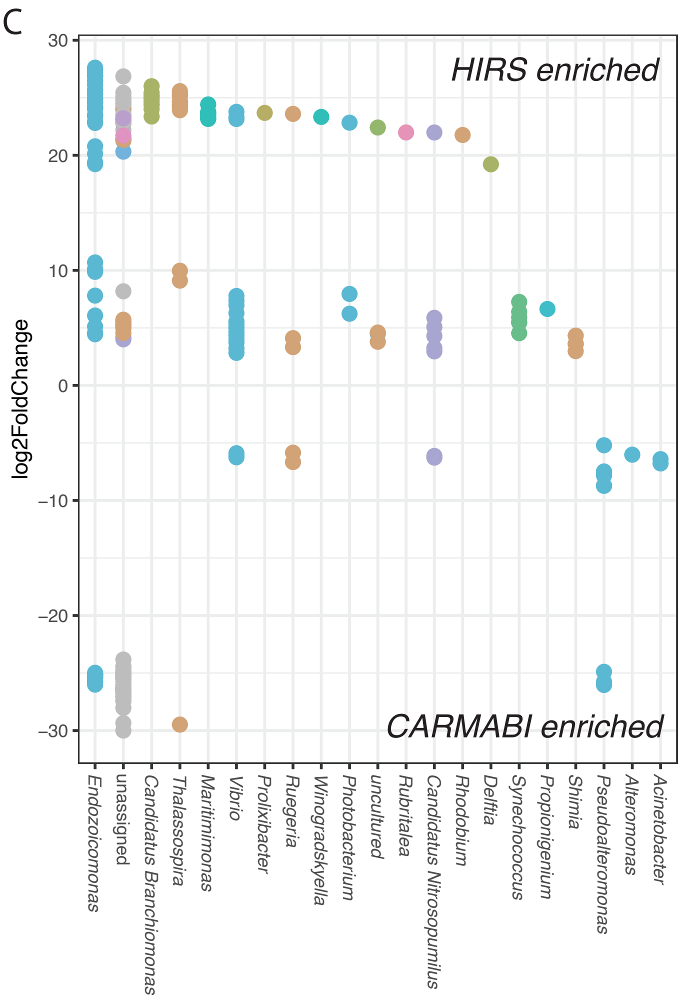

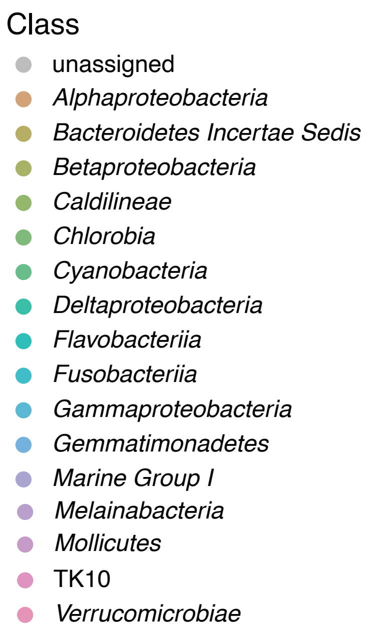

Figure S2. 16S rRNA gene ASVs with differential enrichment between sampling sites. Significant ASVs deter-mined using Wald tests, adjusted p-values < 0.05. A) CARMABI vs HIMB; B) HIMB vs HIRS; C) CARMA-BI vs HIRS. Relative enrichment (log2 scale) shown for ASVs, grouped by assigned genus and colored by microbial class.

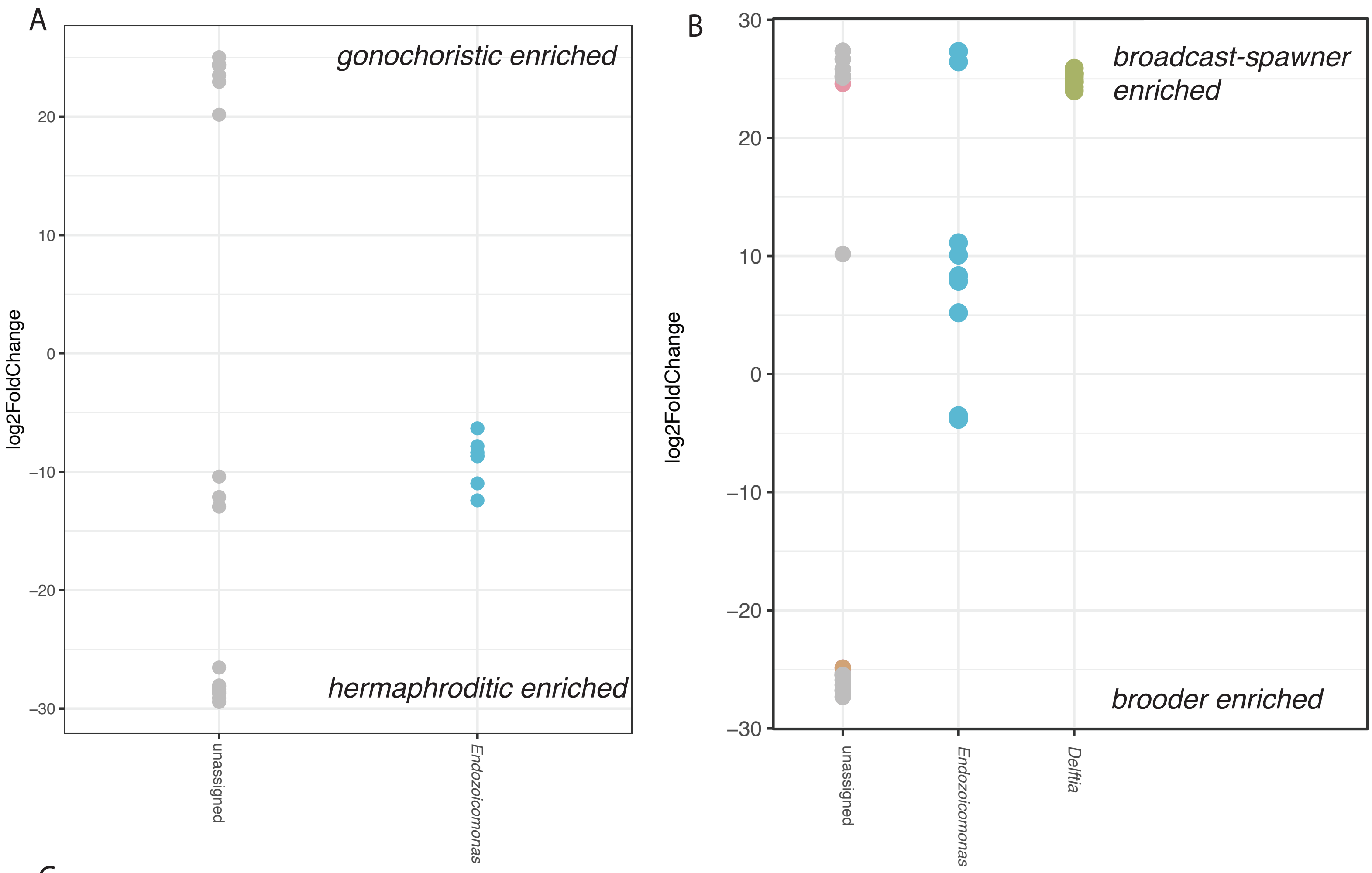

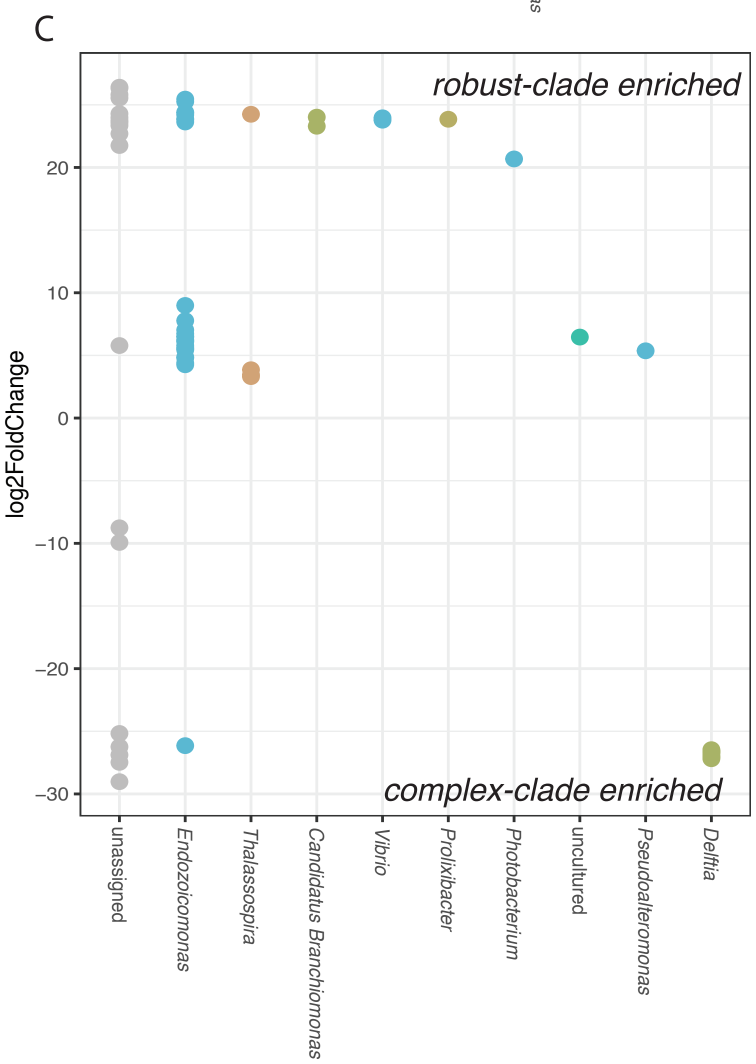

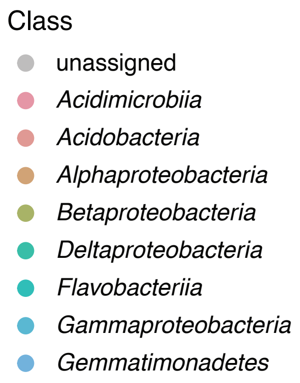

Figure S3. 16S rRNA gene ASVs with differential enrichment with respect to coral life history traits. Significant ASVs determined using Wald tests, adjusted p-values < 0.05. A) reproductive strategy; B) spawning strategy; C) clade. Relative enrichment (log2 scale) shown for ASVs, grouped by assigned genus and colored by microbial class.

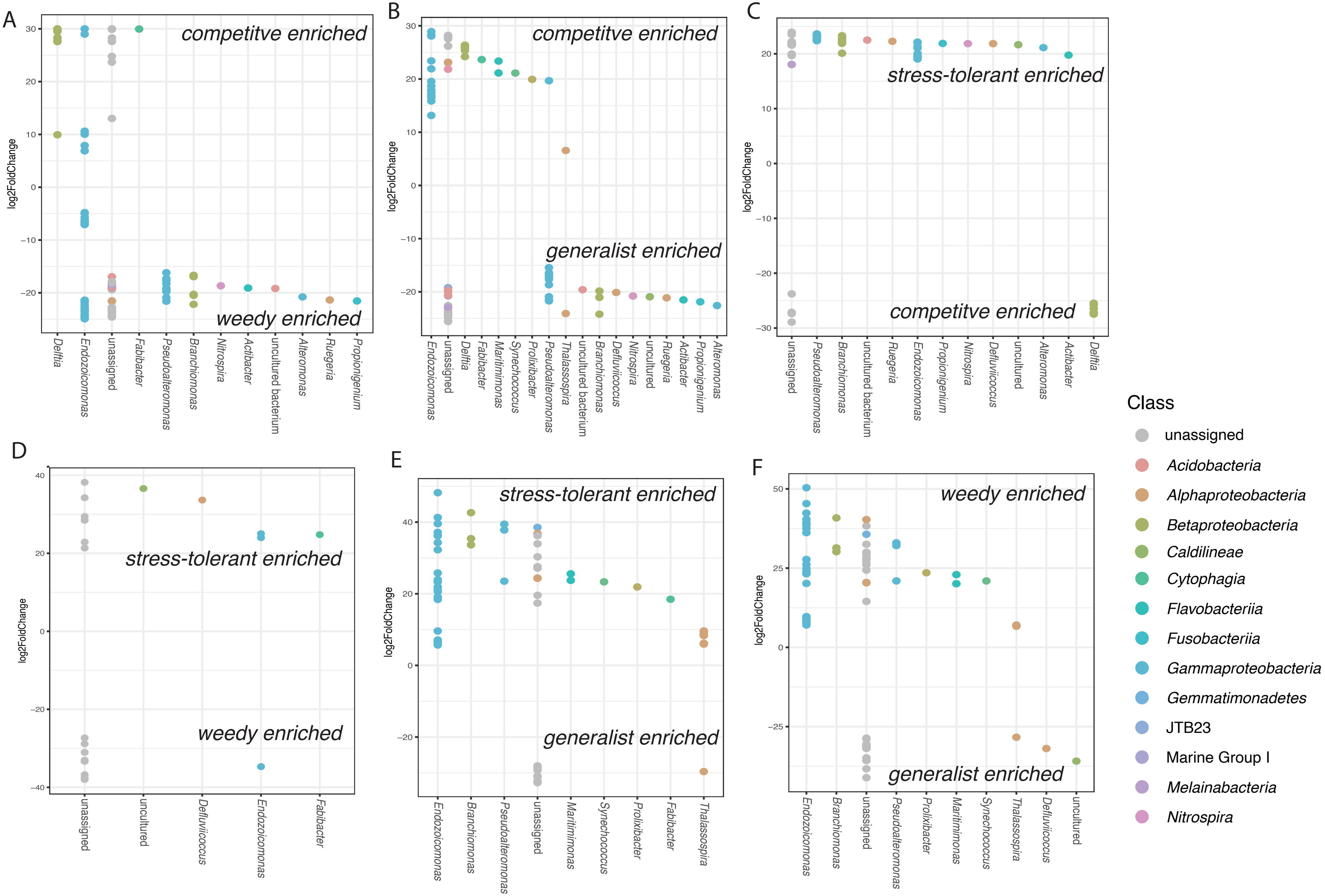

Figure S4. 16S rRNA gene ASVs with differential enrichment with respect to coral ecological life history characteristics. Significant ASVs determined using Wald tests, adjusted p-values < 0.05. A) competitive vs weedy; B) competitive vs generalist; C) competitive vs stress-tolerant; D) weedy vs stress-tolerant; E) generalist vs stress-tolerant; F) weedy vs generalist. Relative enrichment (log2 scale) shown for ASVs, grouped by assigned genus and colored by microbial class.

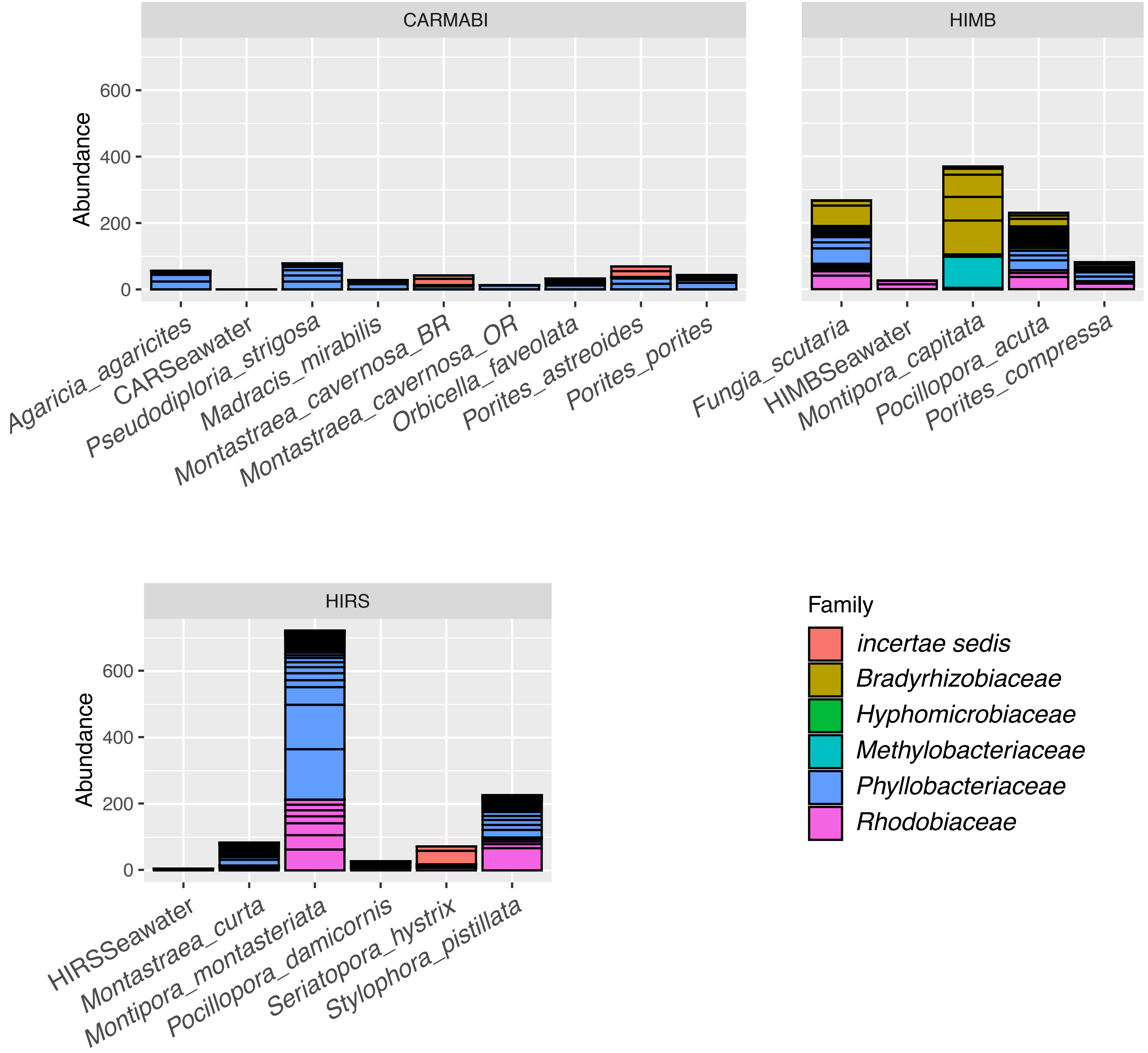

Figure S5. Total counts of 16S rRNA reads (following library normalization) assigned to the order *Rhizobiales* across coral and environmental samples following library size normalization. Abundances are colored according to Family membership.
